# Alanine synthesized by alanine dehydrogenase enables ammonium-tolerant nitrogen fixation in *Paenibacillus sabinae* T27

**DOI:** 10.1073/pnas.2215855119

**Published:** 2022-12-02

**Authors:** Qin Li, Haowei Zhang, Yi Song, Minyang Wang, Chongchong Hua, Yashi Li, Sanfeng Chen, Ray Dixon, Jilun Li

**Affiliations:** ^a^State Key Laboratory for Agrobiotechnology and College of Biological Sciences, China Agricultural University, Beijing 100193, People’s Republic of China; ^b^Department of Molecular Microbiology, John Innes Centre, Norwich NR4 7UH, United Kingdom

**Keywords:** biological nitrogen fixation, ammonium tolerance, glutamine synthetase, alanine dehydrogenase

## Abstract

Most diazotrophs fix nitrogen only under nitrogen-limiting conditions, for example, in the presence of relatively low concentrations of NH_4_^+^ (0 to 2 mM). However, *Paenibacillus sabinae* T27 exhibits an unusual pattern of nitrogen regulation of nitrogen fixation, since although nitrogenase activities are high under nitrogen-limiting conditions (0 to 3 mM NH_4_^+^) and are repressed under conditions of nitrogen sufficiency (4 to 30 mM NH_4_^+^), nitrogenase activity is reestablished when very high levels of NH_4_^+^ (30 to 300 mM) are present in the medium. To further understand this pattern of nitrogen fixation regulation, we carried out transcriptome analyses of *P. sabinae* T27 in response to increasing ammonium concentrations. As anticipated, the *nif* genes were highly expressed, either in the absence of fixed nitrogen or in the presence of a high concentration of NH_4_^+^ (100 mM), but were subject to negative feedback regulation at an intermediate concentration of NH_4_^+^ (10 mM). Among the differentially expressed genes, *ald1*, encoding alanine dehydrogenase (ADH1), was highly expressed in the presence of a high level of NH_4_^+^ (100 mM). Mutation and complementation experiments revealed that *ald1* is required for nitrogen fixation at high ammonium concentrations. We demonstrate that alanine, synthesized by ADH1 from pyruvate and NH_4_^+^, inhibits GS activity, leading to a low intracellular glutamine concentration that prevents feedback inhibition of GS and mimics nitrogen limitation, enabling activation of *nif* transcription by the nitrogen-responsive regulator GlnR in the presence of high levels of extracellular ammonium.

## Significance

Biological nitrogen fixation is a highly energy-intensive process, and thus, nitrogenase synthesis is subject to negative feedback regulation by metabolic signals of the nitrogen status. However, nitrogen fixation in *Paenibacillus sabinae* T27 exhibits a highly unusual response to nitrogen regulation, in which the negative feedback loop is overridden at external concentrations of NH_4_^+^ higher than 30 mM, resulting in ammonia-tolerant nitrogen fixation. We demonstrate that this behavior results from alanine biosynthesis by alanine dehydrogenase, which inhibits glutamine synthetase activity and lowers intracellular glutamine levels with consequent downstream effects on nitrogen fixation gene regulation. Our results suggest a significant potential for engineering ammonium and alanine excretion in plant-associated diazotrophs, thus partially mimicking bacteroid nitrogen metabolism in the rhizobia-legume symbiosis.

Representatives of Bacteria and Archaea (called diazotrophs) utilize nitrogenase to convert atmospheric N_2_ to ammonium in a process known as biological nitrogen fixation. The fixed nitrogen (ammonium) is readily assimilated into amino acids rather than being released into the environment (1). Ammonium assimilation in bacteria is generally achieved through synthesis of glutamate and glutamine from ammonium and 2-oxoglutarate by two pathways. Under nitrogen starvation, the glutamine synthetase (GS)–glutamate synthase (GOGAT) pathway performs ammonium assimilation (2, 3). In this pathway, GS, with a low *Km* for NH_4_^+^ (0.1 mM), catalyzes production of glutamine by incorporating ammonium into glutamate, coupled to GOGAT, which produces two molecules of glutamate by transferring the amide group from glutamine to 2-oxoglutarate. Under excess nitrogen, glutamate dehydrogenase (GDH), with a high *Km* for NH_4_^+^ (1 mM), catalyzes formation of glutamate from ammonium and 2-oxoglutarate. The GS–GOGAT pathway consumes adenosine triphosphate (ATP), while the GDH pathway is energy independent (1). GS is essential for glutamine biosynthesis and is therefore ubiquitous in bacteria.

An additional ammonium assimilation pathway, involving alanine dehydrogenase (called ADH, AlaDH, or AldA), which catalyzes synthesis of alanine from pyruvate and ammonium under high levels of nitrogen excess, is found in some bacteria (4). Similar to GDH, ADH also has a high *Km* value for NH_4_^+^ and does not require ATP (1). Alanine dehydrogenase catalyzes the reductive amination of pyruvate to L-alanine as well as the reverse reaction, the oxidative deamination of L-alanine to pyruvate (NADH + NH_4_^+^ pyruvate === L-alanine + NAD^+^ + H_2_O) (4). Both reductive amination and oxidative deamination by this enzyme play an important role in various physiological processes in bacteria. L-alanine produced by ADH through the reductive amination of pyruvate is required for synthesis of other amino acids, proteins, and the peptidoglycan layer in the cell wall of bacteria (e.g., *Streptomyces aureofaciens* (5) and *Rhodobacter capsulatus* (6)). Alanine dehydrogenase (AldA) has been detected in soybean, alfalfa, pea, and lupin bacteroids (7–11) and is the key enzyme for synthesis of alanine in pea nodule bacteroids that export both alanine and ammonia to the plant (10) and in soybean nodule bacteroids that excrete only alanine (12). However, export of alanine as the sole nitrogen source secreted by bacteroids has been disputed by others (13, 14). The oxidative deamination of alanine to pyruvate by ADH provides energy in the tricarboxylic acid cycle (TCA) for sporulation of *Bacillus subtilis* (15) and for normal diazotrophic growth of the cyanobacterium *Anabaena* sp. strain PCC 7120 (16). In addition, ADH has been shown in *Synechococcus elongatus* PCC 7942 to be required for a proper N-starvation response (17). ADH is required for utilization of alanine as a nitrogen source and to help mycobacteria survive under respiration-inhibitory conditions by maintaining an optimal NADH/NAD^+^ ratio during anaerobiosis (18–20). ADH also has important applications in the food, pharmaceutical, chemical, and environmental industries (4), and *ald* genes encoding ADH have been cloned from different bacteria and overexpressed in *Zymomonas mobilis* and *Escherichia coli* (21–25).

Nitrogen metabolism is well studied in *B. subtilis* as the model organism for low G–C gram-positive bacteria (26). Transcriptional regulation of nitrogen metabolism in *B. subtilis* is mediated by two transcription factors, GlnR and TnrA (27). GlnR represses expression of the *glnRA* operon, which encodes GlnR itself and GS (28, 29), the *tnrA* gene (30), and the urease operon *ureABC* (31). *B. subtilis* GS belongs to the α-subgroup of GS1-type enzymes, which is distinguished from the β-subgroup of *E. coli* and *Salmonella typhimurium* by having regulatory properties (26, 32, 33). The activity of *B. subtilis* GS is feedback-inhibited by its product glutamine as well as by multiple end products of glutamine (Gln) metabolism (e.g., AMP, alanine, glycine) (34). Notably, the GS-Gln feedback-inhibited form of GS (FBI-GS) interacts with and regulates the activities of the transcription factors TnrA and GlnR (28, 29, 35–37). FBI-GS forms a stable complex with TnrA that inhibits the DNA-binding activity of TnrA; in contrast, FBI-GS acts as a chaperone to stabilize GlnR–DNA complexes, through a transient protein–protein interaction (28, 38, 39). Thus, *B. subtilis* GS is an unusual multitasking protein that functions as an enzyme, transcription coregulator, and chaperone.

*Paenibacillus,* formerly included in the *Bacillus* genus, is a large genus of gram-positive, endospore-forming, facultative anaerobic bacteria including non-N_2_-fixing and N_2_-fixing species that can associate with plants. Our previous studies have revealed that *Paenibacillus polymyxa* WLY78 exhibits nitrogenase activity under nitrogen-limiting conditions (40–43) and transcription of the *nif* gene operon comprising nine genes (*nifBHDKENXhesAnifV*) in this bacterium is controlled by GlnR according to nitrogen availability (43). However, another member of this genus, *P*aenibacillus* sabinae* T27, exhibits a very unusual profile of nitrogen fixation in response to the external nitrogen concentration, expressing high levels of nitrogenase activity at both low (0 to 3 mM) and high (30 to 300 mM) concentrations of ammonium but little activity in the medium range (4 to 30 mM NH_4_^+^), which already provides nitrogen sufficiency (44). This unexpected pattern of nitrogen fixation regulation, whereby nitrogenase is expressed and active when it is not required to provide fixed nitrogen to the cell, has not so far been observed in other diazotrophs. In this study, we demonstrate that alanine dehydrogenase (ADH1), encoded by *ald1*, is responsible for nitrogen fixation in the presence of high concentrations of external ammonium and that *ald1* transcription is positively regulated by the alanine-responsive regulator AdeR*.* Intracellular alanine levels increased in proportion to the concentration of NH_4_^+^, dependent on *ald1*. We observe that high levels of alanine synthesis, catalyzed by ADH1, inhibit GS activity, leading to a low level of intracellular glutamine and GlnR-mediated activation of *nif* gene expression at high NH_4_^+^ concentrations. These findings have important implications for nitrogen fixation in natural habitats and may facilitate synthetic approaches to engineer ammonium-tolerant nitrogen fixation.

## Results

### Transcriptome Analysis of *P. Sabinae* T27 in Response to Increasing Ammonium Concentrations.

Our recent study has shown that *P. sabinae* T27 unexpectedly exhibits nitrogen fixation at very high (30 to 300 mM) concentrations of NH_4_^+^, but nitrogenase activity is repressed in the 4 to 30 mM concentration range of ammonium, which represents nitrogen sufficiency (44). To analyze this unusual pattern of nitrogen regulation further, we have performed transcriptome analysis of *P. sabinae* T27 grown anaerobically in three different nitrogen regimes (0 mM, 10 mM, and 100 mM NH_4_^+^, respectively) using high-throughput Illumina sequencing technology. The 10 *nif* genes (*nifBHDKENXorf1hesAnifV*) within the main *nif* cluster of *P. sabinae* T27 were highly expressed under both 0 mM and 100 mM NH_4_^+^ in comparison to those under 10 mM NH_4_^+^ conditions (*SI Appendix*, Fig. S1*BD* and Table S1), consistent with nitrogenase activities in different concentrations of NH_4_^+^ (*SI Appendix*, Fig. S1*A*). However, transcript levels of other nitrogen metabolism genes were also influenced by the ammonium concentration; notably, *ald1*, encoding alanine dehydrogenase (ADH1), was significantly up-regulated at 100 mM NH_4_^+^ (compare the RNA-seq data in *SI Appendix*, Fig. S1*C* with qRT-PCR analysis in *SI Appendix*, Fig. S1*E* and Table S1). Two other genes, *yjeH*, encoding an amino acid transporter, and *PSAB_05205*, encoding a drug/metabolite transporter permease, were also up-regulated in the presence of high (100 mM) ammonium (*SI Appendix*, Table S1 and Fig. S1).

### Mutation of *ald1* Prevents *nif* Gene Expression and Nitrogenase Activity in the Presence of a High Level of NH_4_^+^.

*P. sabinae* T27 has two *ald* genes (named *ald1* and *ald2*), whereby *ald1* is linked together with the *adeR* gene (encoding a transcription regulator), while *ald2* is located elsewhere in the genome. The alanine dehydrogenase (ADH1) enzyme encoded by *ald1* has 63% identity with ADH2 (encoded by *ald2*), and phylogenetic analysis indicates that both ADH1 and ADH2 of *P. sabinae* T27 are closely related to ADH (termed Ald) from *B. subtilis* 168 (*SI Appendix*, Fig. S2) with amino acid sequence similarity between 64% and 71%.

**Fig. 1.**
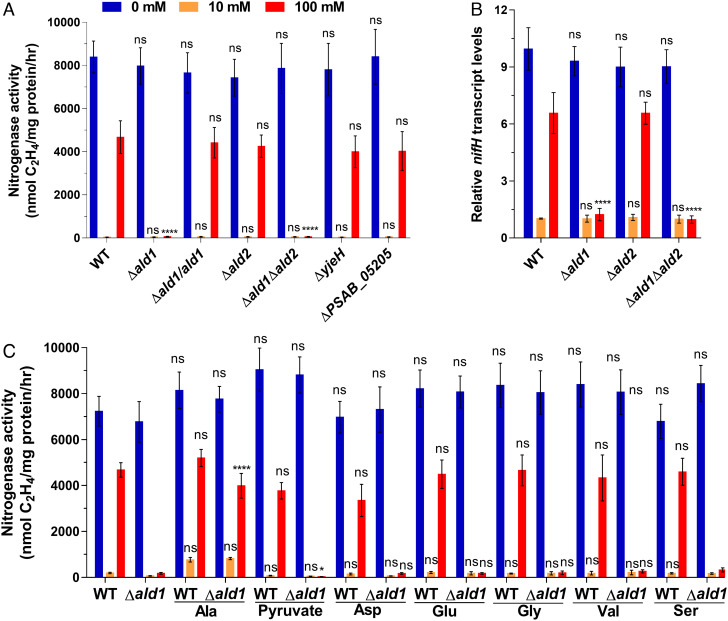
Influence of mutations on *nif* gene expression, nitrogenase activity and suppression of the Δ*ald1* phenotype by exogenous alanine. (*A*) Nitrogenase activities of WT, deletion mutants Δ*ald1*, Δ*ald2*, Δ*yjeH*, Δ*PSAB_05205*, and Δ*ald1*Δ*ald2* and the complemented strains Δ*ald1*/*ald1*. (*B*) qRT-PCR analysis of *nifH* transcripts in WT, Δ*ald1*Δ*ald2*, and Δ*ald1*Δ*ald2* mutants. (*C*) Nitrogenase activity of WT and Δ*ald1* mutant grown in nitrogen-free medium supplemented with 5 mM alanine (Ala), pyruvate, aspartate (Asp), glutamate (Glu), glycine (Gly), valine (Val), and serine (Ser), respectively. Results are representative of at least three independent experiments. Error bars indicate SDs. Statistical analysis was carried out by two-way ANOVA with Tukey's multiple comparisons used to compare means. *P* values are indicated as ns (not significant) *P* > 0.05, *P < 0.05, **P < 0.01, ***P < 0.001, ****P < 0.0001. The WT and Δ*ald1* strains grown in the absence of alanine were used as reference groups for statistical comparisons.

In order to investigate whether the four genes *ald1*, *ald2*, *yjeH*, and *PSAB_05205* are involved in nitrogen fixation when high concentrations of ammonium are available, deletion mutants of Δ*ald1*, Δ*ald2*, Δ*ald1*Δ*ald2*, Δ*yjeH*, and Δ*PSAB_05205* were constructed together with their corresponding complemented strains. The nitrogenase activities of the Δ*ald2*, Δ*yjeH*, and Δ*PSAB_05205* mutants were similar to those of the wild-type (WT) strain irrespective of the ammonium concentration (Fig. 1*A*). However, in contrast to the wild type, the nitrogenase activity of the Δ*ald1* mutant was barely detectable at 100 mM NH_4_^+^. Similarly, the Δ*ald1*Δ*ald2* double mutant lost the ability to exhibit nitrogenase activity in the presence of 100 mM NH_4_^+^ (Fig. 1*A*). Complementation of Δ*ald1* with the *ald1* gene (strain Δ*ald1*/*ald1*) restored nitrogenase activity to the WT level in the presence of high ammonium. These results suggest that *ald1* is uniquely required for nitrogen fixation under high concentrations of NH_4_^+^, rather than *ald2*, *yjeH*, or *PSAB_05205*. qRT-PCR analysis of *nifH* transcripts in the Δ*ald1* and double Δ*ald1*Δ*ald2* deletion mutants supports the conclusion that *ald1* is required to enable *nif* transcription and nitrogenase activity at high NH_4_^+^ concentrations (Fig. 1*B*).

### Alanine Synthesis Catalyzed by ADH1 Is Responsible for Nitrogen Fixation in High NH_4_^+^.

As described above, inactivation of the *ald1* gene resulted in loss of nitrogenase activity under high NH_4_^+^. It is known that ADH catalyzes production of alanine from NH_4_^+^ and pyruvate. Thus, the effects of pyruvate, alanine, and other amino acids (aspartate, glutamate, glutamine, glycine, valine, and serine) on nitrogenase activities of WT and Δ*ald1* mutant strains were determined. Addition of 5 mM alanine led to recovery of 80% nitrogenase activity in the Δ*ald1* mutant under 100 mM NH_4_^+^ (Fig. 1*C*). However, the impaired nitrogenase activity of the Δ*ald1* mutant was not recovered by addition of pyruvate or the other amino acids under high ammonium conditions (Fig. 1*C*). These results suggest that ADH1 is required for nitrogen fixation when *P. sabinae* is grown on 100 mM NH_4_^+^ as a consequence of its ability to synthesize alanine. In agreement with this conclusion, RT-PCR analysis demonstrated that *nifH* transcription was proportional to the level of *ald1* expression in the range 30 to 100 mM NH_4_^+^ (*SI Appendix*, Fig. S3*A*), and qRT-PCR analysis revealed that addition of exogenous alanine suppressed the deficiency in *nifH* transcription in the *ald1* mutant within this concentration range (*SI Appendix*, Fig. S3*B*). Notably, the addition of alanine also significantly increased *ald1* transcription in the WT strain, particularly in the presence of 100 mM NH_4_^+^ (*SI Appendix*, Fig. S3*C*).

**Fig. 2.**
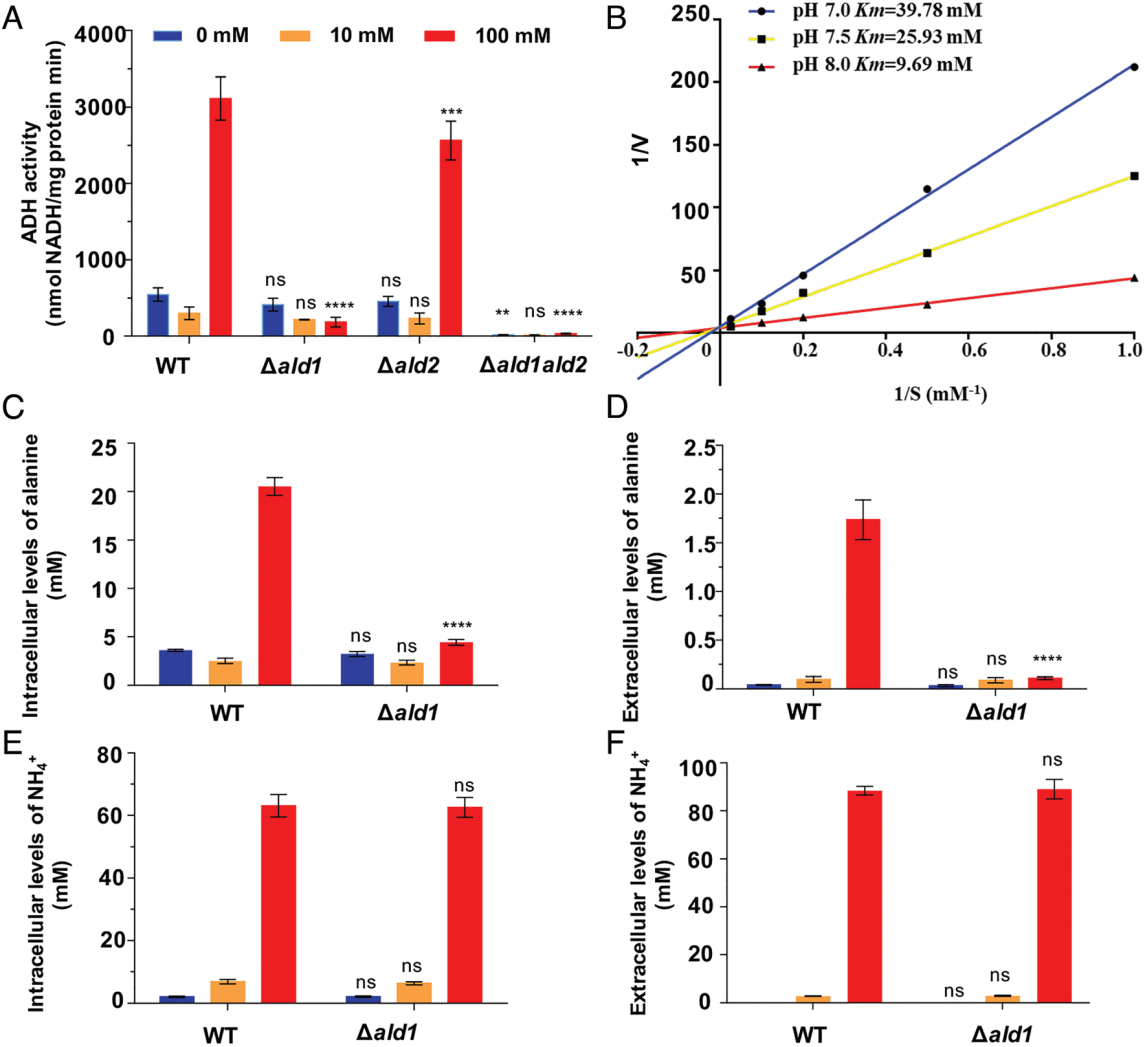
Reductive amination activity of ADH1 and extracellular and intracellular levels of alanine and NH_4_^+^ in WT and mutant strains. (*A*) Reductive amination activity of ADH1 determined in crude extracts of the strains indicated on the x axis grown in the absence or presence of ammonium at concentrations indicated in the legend. (*B*) Double reciprocal plots of the effect of variation of NH_4_Cl concentration on the reductive amination activity of alanine dehydrogenase at three different pH values. (*C* and *D*) Extracellular and intracellular levels of alanine in strains grown with the indicated concentrations of ammonium. (*E* and *F*) Extracellular and intracellular levels of NH_4_^+^. Bars represent mean ± SD (n = 3). Statistical analysis was carried out by two-way ANOVA with Tukey’s multiple comparisons used to compare means. *P* values are indicated as ns (not significant) *P* > 0.05, *P < 0.05, **P < 0.01, ****P < 0.0001. The WT strain was used as the reference group for statistical analysis.

We considered that the reductive amination of pyruvate to L-alanine catalyzed by ADH1 might either a) lower the intracellular NH_4_^+^ concentration to enable derepression of nitrogenase or b) the synthesis of alanine itself could influence nitrogen regulation of nitrogen fixation. The latter seems more likely given that exogenous alanine suppresses the *ald1* phenotype. Crude extracts from cells grown in the absence or presence of 10 mM NH_4_^+^ exhibited basal levels of ADH1-mediated reductive amination, but crude extracts from cells grown with 100 mM NH_4_^+^ exhibited high levels of activity. As anticipated, the enzyme activity in the Δ*ald1* mutant did not increase in the presence of high levels of ammonium and was abolished in the Δ*ald1*Δ*ald2* double mutant, whereas activity in the Δ*ald2* crude extract was similar to the WT levels (Fig. 2*A*). This suggests that *P. sabinae* ADH1 is responsible for alanine synthesis from NH_4_^+^ and pyruvate, and the specific activity of ADH1 is dependent on the NH_4_^+^ concentration under which the cells were grown.

*P. sabinae* ADH1 with a His_6_-tag at the N terminus was overexpressed and purified from *E. coli*. The optimal pH of the enzyme was pH 8.0, and the apparent *Km* values for NH_4_^+^ at pH 7.0, pH 7.5, and pH 8.0 were 39.78 mM, 25.93 mM, and 9.69 mM, respectively, (Fig. 2*B*) potentially reflecting the dissociation constant (p*K_a_*) of NH_4_^+^. This suggests that the capacity of *P.  sabinae* ADH1 to exhibit NH_4_^+^ assimilation in vivo is dependent both on the intracellular concentration of ammonium and the cytoplasmic pH. We measured the extracellular and intracellular concentrations of alanine and NH_4_^+^ in the WT and Δ*ald1* mutant strains grown on different concentrations of ammonium. High-performance liquid chromatography (HPLC) analysis revealed that the intracellular alanine level in the WT strain was 3.6-fold higher than that of the Δ*ald1* mutant when grown on 100 mM NH_4_^+^ (Fig. 2*C*), and the extracellular alanine concentration was 14.7-fold higher than that of the Δ*ald1* mutant when grown under these conditions (Fig. 2*D*). To confirm the increase in intracellular alanine at high ammonium concentrations, we repeated the metabolite analysis using ultrahigh performance liquid chromatography (UHPLC)-high-resolution tandem mass spectrometry (HRMS), which also demonstrated the substantial increase in the alanine concentration at 100 mM NH_4_^+^, which did not occur in the Δ*ald1* mutant (*SI Appendix*, Table S2). Thus, ADH1 is mainly responsible for alanine synthesis from NH_4_^+^ and pyruvate in the presence of 100 mM NH_4_^+^, resulting in alanine excretion (~1.5 μM). In contrast to the intracellular and extracellular alanine levels, there were no obvious differences in the intracellular and extracellular NH_4_^+^ levels between the WT and Δ*ald1* mutant strains (Fig. 2 *E* and *F*).

### AdeR Positively Regulates Transcription of *ald1* at High NH_4_^+^ Concentrations.

As noted above, *ald1* expression increased in WT *P. sabinae* at elevated ammonia concentrations and in response to the addition of exogenous alanine (*SI Appendix*, Fig. S3*C*). In *B. subtilis*, AdeR, the regulator of the *ald* gene, activates *ald* expression in response to alanine availability and is required for sporulation (42). *P. sabinae* AdeR shows 31% similarity with *B. subtilis* AdeR, and both the *ald1* and *adeR* genes are expressed in the same transcriptional orientation in *P. sabinae* T27 (*SI Appendix*, Fig. S4*A*). We identified the transcription start site (TSS) of *ald1* using 5′RACE and a putative AdeR binding site located upstream of the *ald1* promoter (*SI Appendix*, Fig. S4*B*). Electrophoretic mobility shift assays (EMSA) revealed that a His-tagged derivative of *P. sabinae* AdeR, expressed and purified from *E. coli*, specifically binds to the *ald1* promoter region containing the AdeR binding site (*SI Appendix*, Fig. S4 *C* and *D*).

We investigated the role of AdeR and the putative AdeR binding site on *ald1* transcription and its relationship to *nif* gene expression (*SI Appendix*, Fig. S5). *ald1* transcription increased substantially in the WT strain when grown on 100 mM NH_4_^+^. In contrast, expression of *ald1* was ablated in a strain carrying a deletion of *adeR* or in a strain designated MAdeR, which carries a mutation in the putative AdeR DNA binding site (in which the consensus sequence CTCATT is replaced by GGATCC). Notably, in both cases, the deficiency in *ald1* transcription was not suppressed by addition of alanine (*SI Appendix*, Fig. S5*A*). Both the Δ*adeR* and MAdeR mutations prevented *nif* gene expression in the presence of a high level of NH_4_^+^ (100 mM), as anticipated from their influence on *ald1* expression. However, this deficiency could be suppressed by the addition of exogenous alanine, which is expected if alanine, rather than ADH1 itself, is responsible for *nif* transcription at high NH_4_^+^ concentrations (*SI Appendix*, Fig. S5*B*). The influence of the mutations on *nif* transcription was mirrored by their effects on acetylene reduction activity, and we also verified that the Δ*adeR* phenotype could be complemented by a plasmid carrying *adeR* (*SI Appendix*, Fig. S5*C*). These data indicate that AdeR positively regulates *ald1* expression in the presence of excess ammonium in *P. sabinae* T27 and is responsive to alanine availability, consistent with the properties of AdeR in *B. subtilis* (45).

### Alanine Inhibits GS Activity Resulting in Lower Levels of Intracellular Glutamine.

**Fig. 3.**
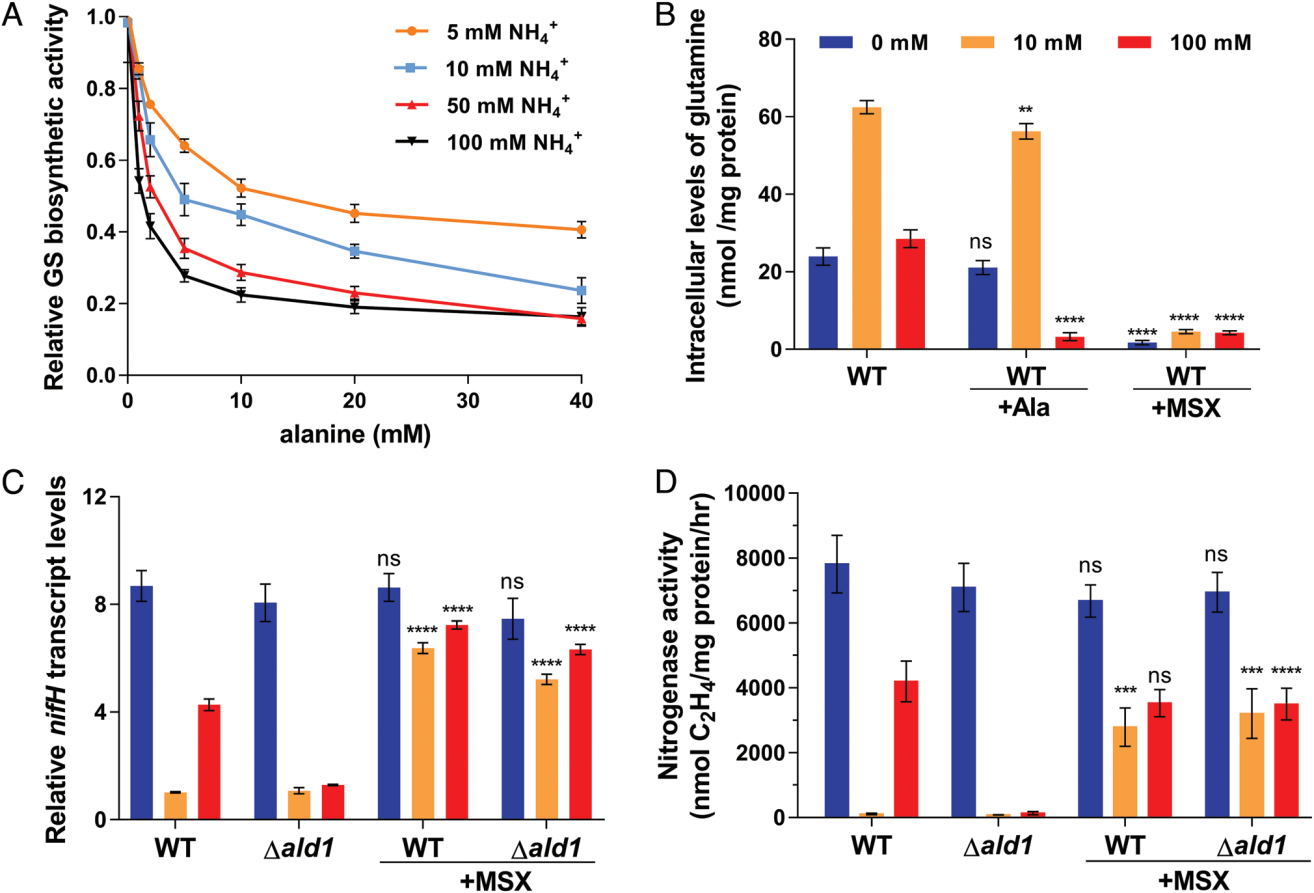
Effect of alanine and MSX on GS activity, *nif* gene expression, and nitrogenase activity. (*A*) Inhibition of GS activity by alanine in vitro in the presence of three different concentrations of ammonium as indicated on the x axis. The reaction solution contained, 5 mM alanine, and 100 mM Na-glutamate. (*B*) Effect of alanine and MSX on the intracellular glutamine content of WT and Δ*ald1* mutant strains. (*C*) qRT-PCR analysis of the influence of MSX on *nifH* transcription of WT and Δ*ald1* mutant strains. (*D*) Effect of MSX on the nitrogenase activity of WT and Δ*ald1* mutant strains. Final concentrations of alanine and MSX in panels *B*–*D* were both 5 mM, respectively. Statistical analysis was carried out by two-way ANOVA with Tukey’s multiple comparisons used to compare means. *P* values are indicated as ns (not significant) *P* > 0.05, *P < 0.05, **P < 0.01, ****P < 0.0001. Cultures without addition of an exogenous compound (alanine or MSX) were used as the reference group for statistical comparisons.

We rationalized that alanine could potentially influence nitrogen regulation of nitrogen fixation via an effect on metabolic signals of nitrogen availability. Since alanine inhibits the activity of GS either by binding to the active site (33) or potentially via an allosteric interaction in the GS1-α class of enzymes (34), we investigated whether *P. sabinae* GS was similarly inhibited by alanine. We overexpressed and purified an N-terminally His-tagged version of *P. sabinae* T27 GS in *E. coli* and observed that alanine inhibited GS biosynthetic activity at ammonia concentrations of 10 mM and above (Fig. 3*A*), consistent with previous reports that alanine is an effective inhibitor of *B. subtilis* GS in vitro when NH_4_^+^ is present at saturating (50 mM), rather than limiting concentrations (34). We anticipated that inhibition of GS by alanine would result in decreased levels of intracellular glutamine in vivo. In the WT strain, glutamine levels increased considerably in conditions of N sufficiency (10 mM NH_4_^+^) compared with nitrogen limitation (0 mM NH_4_^+^) as expected from an increased rate of ammonium assimilation by GS under nitrogen-sufficient conditions. However, the intracellular glutamine concentration decreased to a level similar to that observed under nitrogen-limiting conditions when the WT strain was grown in the presence of 100 mM NH_4_^+^ (Fig. 3*B* and *SI Appendix*, Table S2), and this decreased even further when exogenous alanine was also added to the WT (Fig. 3*B*) or to the *ald1* mutant strain (*SI Appendix*, Table S2). These results support the inference that alanine inhibits GS activity in vivo, especially under high concentrations of external NH_4_^+^ when the intracellular concentration of ammonium is likely to exceed the *Km* for ADH (Fig. 3*B*), resulting in high levels of alanine synthesis (*SI Appendix*, Table S2). However, in contradiction to the results obtained in the presence of 100 mM NH_4_^+^, we did not observe major decreases in the glutamine level when exogenous alanine was added to either the WT strain (Fig. 3*B*) or the *ald1* strain (*SI Appendix*, Table S2) grown in the presence of 10 mM NH_4_^+^. This was also reflected in the glutamine to 2-oxoglutarate ratio, which did not reduce to the level observed when exogenous alanine was added to the *ald1* mutant strains grown in 100 mM NH_4_^+^. Although intracellular alanine levels increased when exogenous alanine was added to cultures of the *ald1* mutant, they did not exceed the level observed in the WT strain when grown with 10 mM NH_4_^+^ (*SI Appendix*, Table S2). We therefore posit that the inability of exogenous alanine to suppress negative feedback regulation of nitrogen fixation of either the WT or the *ald1* mutant in the presence of 10 mM NH_4_^+^ (Fig. 1*C*) reflects the relatively low intracellular concentration of alanine under these conditions, which in combination with the relatively low level of intracellular ammonium (Fig. 2*E*) does not permit inhibition of GS activity.

Since we were unable to obtain a *glnA* deletion mutant of *P. sabinae* T27, which could be an essential gene, methionine sulfoximine (MSX), a GS activity inhibitor was used to investigate the effect of GS activity on *nif* gene expression and nitrogenase activity. After treatment with MSX, *P. sabinae* T27 exhibited very low levels of intracellular glutamine under all NH_4_^+^ conditions, implying that GS is the only enzyme responsible for glutamine synthesis (Fig. 3*B*). MSX treatment led to a significant increase in *nifH* transcription in both WT and Δ*ald1* mutant strains in 10 mM NH_4_^+^ and also led to recovery of *nif* gene expression in the Δ*ald1* mutant in 100 mM NH_4_^+^ (Fig. 3*C*). Similar increases in nitrogenase activity were observed after MSX treatment under these conditions (Fig. 3*D*). Notably, in the absence of MSX, the glutamine to 2-oxoglutarate ratio in the WT strain was highest under 10 mM NH_4_^+^ (*SI Appendix*, Table S2), consistent with a negative correlation between the glutamine level and nitrogen fixation and consequent feedback regulation of nitrogenase expression in the presence of 10 mM NH_4_^+^. Overall, these results are conversant with our recent finding in *P. polymyxa* WLY78 that deletion of *glnA* leads to constitutive expression of *nif* genes under excess nitrogen conditions (43) and implicate glutamine as a metabolic signal for nitrogen regulation of nitrogen fixation in *Paenibacillus.*

### Involvement of GlnR-Binding Sites in Positive and Negative Regulation of *nif* Transcription.

The absence of nitrogen regulation of nitrogenase expression at high concentrations of ammonium is likely to reflect altered regulation of the *P. sabinae* T27 *nif* operon (44). Two GlnR-binding sites, with the consensus sequence 5′-TGTNAN7TNACA-3′, were predicted in the promoter region of the operon, separated by 119 bp (*SI Appendix,* Fig. S6*A*). Site I is located upstream of the −35 region, and site II is positioned downstream of the −10 region of the *nifB* promoter. EMSA with purified *P. sabinae* T27 GlnR protein demonstrated binding of GlnR to the two sites in vitro (*SI Appendix*, Fig. S6*B*). Surface plasmon resonance (SPR) experiments revealed that GlnR alone could specifically bind to the two GlnR-binding sites, and the affinity of GlnR for site I is significantly higher than for site II (*SI Appendix*, Fig. S6 *C* and *D*).

Site-specific mutagenesis of the two GlnR-binding sites was performed by replacing the half-site consensus TGACAT in either site with different restriction enzyme cleavage sites introduced into *P. sabinae* T27 genome via homologous recombination (*SI Appendix*, Fig. S7*A*). Mutation of GlnR-binding site I (mutant MR1) resulted in a low level of *nifH* transcription and resultant loss of nitrogenase activity irrespective of the ammonium concentration (compare panels *B* and *C* in *SI Appendix*, Fig. S7*A*). In contrast, mutation of GlnR-binding site II (in strain MR2) resulted in constitutive expression of *nifH* and nitrogenase activity, with substantial loss of nitrogen regulation. Mutation of both GlnR binding sites in the double mutant strain MR3 resulted in loss of nitrogen fixation, similar to the phenotype of the MR1 mutant (*SI Appendix*, Fig. S7 *B* and *C*). We therefore conclude that site I is involved in activating *nif* gene transcription under nitrogen limitation, and site II is involved in *nif* gene repression under excess nitrogen conditions, similar to the regulatory mechanism identified in *P. polyxyma* WLY78 (43).

Repression of *nif* gene expression in both *P. riograndensis* and *P. polyxyma* is facilitated by the interaction of FBI-GS with GlnR, which stabilizes the binding of GlnR to *nif* promoters (43, 46). We anticipated that a similar interaction between FBI-GS and GlnR occurs in *P. sabinae* T27 to prevent rapid dissociation of GlnR from binding site II. EMSA experiments (*SI Appendix*, Fig. S8) revealed that the affinity of GlnR for this DNA binding site was increased by adding a mixture of Gln and GS (to form FBI-GS), consistent with the role of FBI-GS in stabilizing GlnR-DNA complexes in other *Paenibacillus* strains (43, 46). To determine whether this chaperone function of FBI-GS is perturbed in the presence of alanine, we added both glutamine and alanine to the binding reaction in addition to GS, GlnR, and the DNA fragment carrying binding site II. However, alanine did not apparently decrease the affinity of GlnR for DNA in the presence of FBI-GS, implying that high concentrations of alanine do not lead to derepression of *nif* gene expression by a mechanism that involves disruption of the interaction between FBI-GS and GlnR (*SI Appendix*, Fig. S8).

## Discussion

**Fig. 4.**
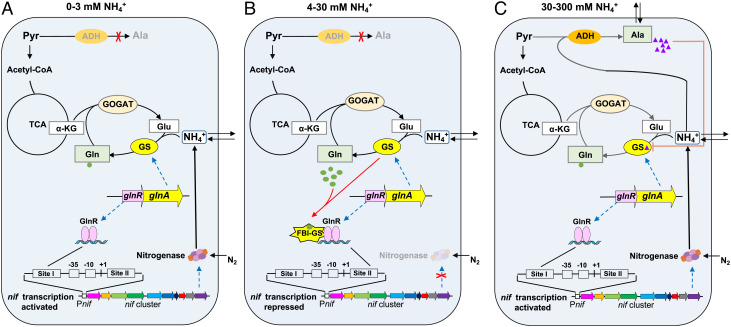
Regulatory model for *nif* gene expression in *P. sabinae* T27 in response to the fixed nitrogen concentration. (*A*) Under nitrogen-limiting conditions (0 to 3 mM NH_4_^+^), the intracellular glutamine content is relatively low and GlnR binds to GlnR-binding site I (located upstream of the −35 region of the *nifB* promoter) to activate *nif* transcription. (*B*) Under medium concentrations of NH_4_^+^ (4 to 30 mM), the intracellular glutamine content is high, resulting in the FBI-GS, which interacts with GlnR thus stabilizing the binding of GlnR to site II (located downstream of the −10 region of the *nifB* promoter), consequently repressing *nif* transcription. (*C*) Under high concentrations of NH_4_^+^ (30 to 300 mM), ADH1 catalyzes conversion of pyruvate and NH_4_^+^ to alanine, which inhibits GS activity and lowers the intracellular glutamine concentration, resulting in GlnR-mediated activation of *nif* gene expression from site I.

The unusual pattern of *nif* gene expression in response to high concentrations of fixed nitrogen in *P. sabinae* T27 is counter-intuitive, as there is no apparent necessity to enable the highly energy-demanding process of nitrogen fixation, when external ammonium is extremely plentiful. Our studies reveal that the reductive amination activity of ADH1, which is induced in response to increases in the intracellular ammonium concentration, is key to enabling ammonia-tolerant nitrogen fixation, as a consequence of increased alanine synthesis. ADH1 may play a role in maintaining NADH/NAD^+^ homeostasis as demonstrated for *Mycobacterium smegmatis* (47), and although wasteful in terms of energy, nitrogen fixation could further serve to remove surplus reductant. A model summarizing our findings, which involves the crucial roles of glutamine, as a metabolic sensor of the nitrogen status, and alanine, as an inhibitor of the GS reaction, is shown in Fig. 4. Essential features of the model under nitrogen deficient conditions (panel *A*) and medium concentrations of ammonium (panel *B*) are highly similar to those reported previously for *P. polymyxa* WLY78 (43). Under nitrogen-limiting conditions, when the flux through the GS–GOGAT nitrogen assimilation pathway is reduced, and intracellular glutamine levels are consequently low, GlnR binds to site I in the *nifB* promoter and activates transcription (Fig. 4*A*). Under nitrogen-excess conditions, when the intracellular glutamine level increases, a pivotal feature in the mechanism is feedback inhibition of GS by glutamine, resulting in FBI-GS, which interacts with the C-terminal tail of GlnR and stabilizes the binding of GlnR to site II in the *nifB* promoter, with consequent repression of *nif* gene transcription (Fig. 4*B*). At high concentrations of ammonium, when the *Km* for ADH1 in *P. sabinae* is likely to be exceeded, and autoactivation of *ald1* expression by AdeR occurs, the intracellular level of alanine substantially increases. Our data suggest that under these conditions, alanine acts as an inhibitor of GS, and as a consequence of this inhibition, the intracellular concentration of glutamine decreases (Fig. 4*C*), as demonstrated by the inverse correlation observed between alanine and glutamine levels (*SI Appendix*, Table S2). The resultant reduction in the glutamine concentration decreases formation of FBI-GS, enabling GlnR to bind to site I in the *nifB* promoter, thus activating *nif* gene expression (Fig. 4*C*). The unexpected tolerance of nitrogen fixation to high levels of ammonia is therefore an outcome of alanine biosynthesis from NH_4_^+^ and pyruvate by ADH1.

Substantial alanine biosynthesis by ADH1 is likely to be triggered by activation of *aldA1* expression by AdeR in the presence of alanine, in conjunction with increases in the intracellular concentration of ammonium above the *Km* for the enzyme. In general, ADH has a relatively high *Km* for ammonium between 20 and 300 mM (8). The *P. sabinae* enzyme has a *Km* for NH_4_^+^ in the lower end of this range (~9 to 40 mM dependent on the pH, with optimal catalytic activity at pH 8.0). This is similar to the kinetic properties of alanine dehydrogenase from the diazotrophic cyanobacterium *Anabaena cylindrica*, which has an optimal pH of 8.0 for the aminating reaction, with *Km* values for ammonium varying between 8 mM at pH 8.7 or above and 133 mM at pH 7.3 (48).

In addition to the elevated level of intracellular alanine present in cultures grown on 100 mM NH_4_^+^, we also observed *ald1*-dependent alanine secretion under these conditions (in the μM range). Given that alanine is a generic inhibitor of GS activity, it is relevant to consider how elevated levels of alanine may influence nitrogen metabolism in other nitrogen-fixing organisms. Alanine excretion by *P. sabinae* parallels, to a certain extent, metabolism in the rhizobium–legume symbiosis, whereby legume nodule bacteroids not only provide NH_4_^+^ but also have been shown to secrete alanine as a nitrogen source for the plant (49, 50). The predominant source of alanine in soybean, pea, and *Mesorhizobium loti* is alanine dehydrogenase (AldA) (13). The AldA enzyme in *B. japonicum* and *R. leguminosarum* bacteroids has a relatively low *Km* for NH_4_^+^ (5 to 9 mM) (9, 10). This is conversant with the apparent concentration of ammonium (12 mM) in soybean bacteroids estimated from leakage rates (51), suggesting that the ammonium concentration in the bacteroid cytosol is sufficient to promote alanine synthesis by AldA (10). Mutation of *aldA* results in barely detectable levels of alanine secretion but does not influence nitrogen fixation by pea bacteroids. However, the *aldA* mutation conferred a significant decrease in the dry weight of pea plants as a consequence of lower total nitrogen secretion (10). A similar phenotype was observed when *aldA* was overexpressed, with no apparent effect on nitrogen fixation, but a detrimental effect on nitrogen acquisition by the plant was demonstrated (52). These results point to the crucial role of alanine in sustaining bacteroid metabolism, and, in particular, the requirement to balance carbon allocation and maintain redox homeostasis under oxygen-limiting conditions (53, 54). In the light of our findings, it would be interesting to determine whether alanine plays an additional metabolic role in regulating bacteroid GS activity as this could further serve to decrease ammonia assimilation by bacteroids. In this case, *aldA* mutations would be expected to enhance the flux through the GS–GOGAT pathway, which could potentially restrict nitrogen transfer to the plant.

The ability of *P. sabinae* T27 to fix nitrogen in the presence of high ammonium concentrations may be restricted to a subgroup of diazotrophic *Paenibacillus* species since it is not observed, for example, in *P. polyxyma* WLY78, which exhibits nitrogenase activity only under nitrogen-limiting conditions (43, 44). This may relate to differences in regulation of expression of *ald1* and the characteristics of the ADH enzymes in the two species since although alanine dehydrogenase is competent to catalyse alanine formation in ammonium grown cells of *P. polymyxa*, ^15^N labeling experiments demonstrated that alanine transaminase provides the major route for alanine biosynthesis under these conditions (55). The presence of distinct lineages among nitrogen-fixing species of *Paenibacillus* is indicative of a separate evolutionary history, and it is notable that *P. sabinae* T27 and *P. polyxyma* WLY78 were isolated from different plant rhizospheres (41).

Engineering plant-associated diazotrophic bacteria to excrete fixed nitrogen and thus help to satisfy the N requirements of cereals crops is an important goal of agricultural biotechnology (1, 56, 57). Although nitrogen signaling networks have diverse mechanistic features and components in different bacterial phyla, a generic feature of nitrogen sensing involves the use of glutamine as an intracellular metabolic signal of the nitrogen status. Hence, mutations that influence GS activity have been shown to influence nitrogen regulation of *nif* gene expression in diverse diazotrophs. The ability of alanine, synthesized by ADH1, to inhibit GS and thus mimic nitrogen deficiency by lowering the glutamine concentration in *P. sabinae* T27 has potential implications for engineering combined ammonia and alanine secretion in plant-associated diazotrophs. ADH might therefore provide a synthetic module not only to prevent negative feedback regulation of *nif* gene expression but also enable ammonia excretion by decoupling nitrogen fixation from ammonia assimilation. We note that the specific activity of the *P. sabinae* ADH1 enzyme (~3 μmol per min. mg protein) is similar to that of the soybean bacteroid enzyme (~1 μmol per min. mg protein), suggesting that there is the potential for N delivery to plants, provided that the *Km* of the enzyme is compatible with intracellular ammonium concentrations derived from constitutive nitrogen fixation. Although *ald1* is expressed only at very high ammonium concentrations in *P. sabinae*, expression of ADH could be brought under the control of plant-specific signals in order to activate ammonia and alanine secretion when bacteria are specifically colonizing plant roots (58).

## Materials and Methods

### Strains, Plasmids, and Growth Conditions.

A summary of strains and plasmids used in this study is provided in *SI Appendix*, Table S4. *P. sabinae* T27 and its derivatives were routinely grown in LD medium (per liter contains: 2.5 g NaCl, 5 g yeast, and 10 g tryptone) at 30 °C for 14 h with shaking at 200 rpm. For assays of nitrogenase activity, *P. sabinae* strains were grown in a nitrogen-limited medium supplemented with 0 to 400 mM NH_4_Cl under anaerobic condition. To measure the growth, strains were grown in a nitrogen-limited medium supplemented with 10 mM NH_4_Cl or 10 mM alanine. The nitrogen-limited medium contained (per liter) 10.4 g Na_2_HPO_4_, 3.4 g KH_2_PO_4_, 26 mg CaCl_2_·2H_2_O, 30 mg MgSO_4_, 0.3 mg MnSO_4_, 36 mg ferric citrate, 7.6 mg Na_2_MoO_4_·2H_2_O, 10 mg p-aminobenzoic acid, 5 µg biotin, 2 mM glutamate, and 4 g glucose as the carbon source. *E. coli* strains JM109 and BL21 were used for routine cloning and expression hosts. Thermosensitive vector pRN5101 was used for gene disruption and complementation experiments in *P. sabinae* T27. When appropriate, antibiotics were added at the following concentrations: 100 μg/mL ampicillin, 12.5 μg/mL tetracycline, and 5 μg/mL erythromycin for maintenance of plasmids. MSX was used on whole cells at a concentration of 1 mM (Sigma-Aldrich, St. Louis, MO, USA).

### Bacterial RNA Extraction.

*P. sabinae* T27 and its derivatives were anaerobically grown in a nitrogen-deficient medium supplemented with different concentrations of ammonium in 250-mL flasks with shaking at 250 rpm for 8 h at 30 °C. The cultures were quickly collected by centrifugation at 4 °C under anaerobic conditions and stored in liquid nitrogen for further use. This experiment was repeated three times. For bacterial RNA extraction, bacterial cultures at each experimental time point were harvested and rapidly frozen in liquid nitrogen. Total RNAs were extracted with RNAiso Plus (TaKaRa, Japan) according to the manufacturer’s protocol. Removal of genomic DNA and synthesis of cDNA were performed using the PrimeScript RT reagent kit with gDNA Eraser (TaKaRa, Japan). The concentration of purified RNA was quantified on a NanoDrop ND-1000 spectrophotometer (NanoDrop Technologies, Thermo Fisher Scientific, USA).

### Transcriptome Analysis.

For transcriptomic analysis, *P. sabinae* T27 was anaerobically grown in a nitrogen-deficient medium supplemented with three different concentrations of ammonium (0 mM, 10 mM, and 100 mM NH_4_Cl) in 250-mL flasks with shaking at 250 rpm for 8 h at 30 °C. RNA was extracted as described above. Illumina HiSeq 4000 sequencing from the total RNA was completed at Majorbio Bioinformatics Technology Company (Beijing, China) according to a default Illumina stranded RNA protocol. The raw reads are archived in the NCBI Sequence Read Archive (SRA) database (with accession numbers SRR20508450, SRR20508451, and SRR20508452). Differential expression analysis of the three ammonium concentrations (two biological replicates per condition) was performed using the DESeq R package (1.18.0) (59). The resulting *P* values were adjusted using the Benjamini and Hochberg approach for controlling the false-discovery rate. Differences in the transcript level with an adjusted *P* value of <0.05 and a log_2_ fold-change >two were considered to be significant, and the genes were assigned as differentially expressed genes (DEGs). For a comparative analysis of the expression levels of different genes, data were normalized as fragments per kilobase of transcript per million reads mapped (FPKM). Transcript profiles of *P. sabinae* T27 grown in three different concentrations of ammonium (0 mM, 10 mM, and 100 mM NH_4_^+^) are shown in *SI Appendix*, Table S1.

### qRT-PCR Analysis.

Quantitative real-time RT-PCR (qRT-PCR) was performed on an Applied Biosystems 7500 Real-Time System (Life Technologies), and results were detected by the SYBR Green detection system with the following program: 95 °C for 15 min, 1 cycle; 95 °C for 10 s and 65 °C for 30 s, 40 cycles. Primers used for qRT-PCR are listed in *SI Appendix*, Table S5. The relative expression level was calculated using the threshold cycle (ΔΔCt) method (60), and 16S rRNA was set as the internal control. Triplicate assays using RNAs extracted in three independent experiments were performed for each target gene.

### Construction of Δ*ald1*, Δ*ald2*, Δ*yjeH*, Δ*PSAB*_*05205*, Δ*ald1*Δ*ald2*, and Δ*adeR* Mutants and Their Complementation Strain.

The in-frame-deletion mutants Δ*ald1*, Δ*ald2*, Δ*yjeH,* Δ*PSAB_05205*, Δ*ald1*Δ*ald2*, and Δ*adeR* were constructed via homologous recombination using the suicide plasmid pRN5101 as described previously (43). The upstream and downstream fragments flanking the coding region of *ald1, ald2, yjeH, PSAB_05205*, and *adeR* were PCR-amplified from the genomic DNA of *P. sabinae* T27, respectively. The primers used for these PCR amplifications are listed in *SI Appendix*, Table S5. The upstream and downstream fragments of five genes were then fused with *Bam*HI/*Hind*III-digested vector pRN5101 in Gibson assembly master mix (New England Biolabs), generating the five recombinant plasmids. Then, each of these recombinant plasmids was transformed into *P. sabinae* T27, and the single-crossover transformants were screened for erythromycin resistance (Em^r^). Subsequently, marker-free deletion mutants (the double-crossover transformants) were selected from the initial Em^r^ transformants after several rounds of nonselective growth at 39 °C. The marker-free deletion mutants were confirmed by PCR amplification and DNA sequencing analysis. The double *ald* deletion mutants were constructed via the same method in the single *ald* deletion mutant background.

Complementation of Δ*ald1* and Δ*adeR* was performed using the corresponding WT DNA fragments containing the coding regions and promoters of their respective genes, which were PCR-amplified from the genomic DNA of *P. sabinae* T27. The respective DNA fragments were then ligated to pRN5101 and then transformed into the corresponding mutants, generating the *ald1* and *adeR* complemented strains Δ*ald1/ald1* and Δ*adeR/adeR*, respectively.

### Acetylene Reduction Assays for Nitrogenase Activity.

For nitrogenase activity assays, *P. sabinae* T27 and their derivatives were individually grown overnight in 50 mL liquid LD media for 16 h at 30 °C with shaking at 200 rpm. The culture was collected by centrifugation, and the pellet was washed three times with sterilized water and then resuspended in a 26-mL sealed tube containing 4 mL nitrogen-limited medium to a final OD_600_ of 0.3 to 0.5. The headspace in the tube was then evacuated and replaced with argon gas (43). Acetylene (10% of the headspace volume) was then injected into the test tubesand the cultures were incubated at 30 °C for 2 to 4 h with shaking at 200 rpm. After incubation,100 μL of gas was withdrawn through the rubber stopper with a gas-tight syringe and manually injected into the gas chromatograph HP6890 to quantify ethylene production. The nitrogenase activity was expressed in nmol C_2_H_4_/mg protein/hr.

### Enzyme Assays.

The cells were harvested at the same stage as those taken for qRT-PCR analysis by centrifugation at 10,000 × g for 10 min, washed twice in ice-cold corresponding enzyme activity assay buffers, and then resuspended in the same buffer solution (ca. 15 g wet cells in 50 mL buffer solution). Cell disruption was achieved by sonication, and the resulting crude cell extracts were immediately used for the measurements of enzyme activities or stored at −80 °C in aliquots. All the above mentioned operations were carried out on ice (61). In the present study, enzyme activities involved in the N-assimilation pathway were measured using a temperature controlled spectrophotometer at 37 °C. The protein concentrations were estimated by the Bradford assay method. Each enzyme was measured three times from the same culture.

GS biosynthetic activity was assayed by a modification of the radiochemical method to measure phosphate release (62). The assay solution contained the following materials in a final volume of 460 µL: 100 mM imidazole-HCl buffer (pH 7.0), 50 mM NH_4_Cl, 50 mM Na-glutamate, 10.0 mM ATP, and 20 mM MnCl_2_. The reaction was initiated by addition of 100 μL cell-free extract. After incubation for 30 min at 25 °C, the reaction was stopped by the addition of 1.8 mL FeSO_4_ 7H_2_O solution (0.8% w/v in 0.0075 M H_2_SO_4_), and the tubes were vortexed and placed on an ice bath. Thereafter, 0.15 mL of the color-forming reagent ammonium molybdate (6.6% w/v in 3.75 M H_2_SO_4_) was added. The reaction tubes were again vortexed vigorously, placed on an ice bath, and immediately read at 850 nm. Biosynthetic GS activity was quantified in terms of nm PO_4_^3-^/assay.

For alanine dehydrogenase activity (ADH), the reaction mixture contained the following in a 1-mL volume: 100 mM Tris-HCl (pH 8), 1 mM Na-pyruvate, 133 mM NH_4_Cl, 100 µM NADH, and cell extract containing 0.1 to 0.5 mg protein. Reactions were started by addition of NADH, and the mixture was incubated at room temperature (about 25 °C). Oxidation of NADH was monitored following the decrease in A340 for 5 min. Activity is expressed as nmol of NADH oxidized per mg of protein per min. Nonspecific oxidation of NADH was determined from reactions lacking ammonia. One unit of activity was defined as the amount needed to convert 1 nmol of NADH per mg of protein per min to NAD.

### Quantification of Alanine, Glutamine, Glutamate, Pyruvate, and NH_**4**_^+^.

*P. sabinae* cells were grown anaerobically under different ammonium conditions for 10 h. Then, 100 mL culture was harvested by centrifugation at 5,000 × g for 5 min at 4 °C, and the supernatant was stored at −20 °C for determination of the extracellular content of alanine. The cell pellet was washed with double-distilled H_2_O, resuspended in 2 mL ice-cold water, and disrupted by sonication on ice. The cell-free crude extract was obtained by centrifugation at 20,000 × g for 15 min at 4 °C. Then, the supernatant was collected and stored at −20 °C for determination of intracellular content of alanine. Alanine concentrations were determined with HPLC. The alanine was first derivatized by DNFB (2,4-dinitrofluorobenzene) prior to analysis (63). Analysis was performed by HPLC equipped with a C18 column (Agilent, 5 μm, 4.6 × 150 mm) and a diode array detector at 360 nm. The mobile phase consisted of 35% solution A (acetonitrile) and 65% solution B (1‰ formate in ddH_2_O) at a flow rate of 1.0 mL/min. The chromatographic separation was performed at 40 °C. The above analysis methods were all experimentally verified with standard alanine. The compound concentrations in samples were quantitatively determined with the calibration curve using linear regression.

Pyruvate concentrations were determined by using the colorimetric assay kit from Solarbio Life Sciences (Beijing, China). The assay principle is based on the fact that pyruvate reacts with 2,4-dinitrophenylhydrazine to produce pyruvic acid-2,4-dinitrophenylhydrazone, which shows color in an alkaline solution.

For glutamine and glutamate quantifications, *P. sabinae* cultures (2 mL) were harvested by centrifugation at 14,000 g for 5 min and immediately frozen in liquid nitrogen. Cell pellets were resuspended in 500 μL deionized water. Cells were disrupted by one cycle of sonication (7 W, 50 s; ultrasonic homogenizer, model 3000, Biologics, Inc., Cary, NC, USA) and filtered through a centrifugal filter (Amicon Ultra 30K; Millipore, Billerica, MA, USA). Glutamine concentrations were determined using the glutamine colorimetric assay kit (BioVision Incorporated, Milpitas, CA, USA). Glutamate concentrations were determined using the glutamate colorimetric assay kit from BioVision Incorporated.

For measuring extracellular NH_4_^+^ concentrations, 1 mL culture broth was taken and centrifuged for 10 min at 12,000 rpm to obtain cell-free supernatant. Afterwards, 500 μL of the supernatant was stored at −20 °C for further use. The NH_4_^+^ concentration was determined by using the indophenol method (64).

### Metabolite Analysis by UHPLC-HRMS/MS.

The intracellular concentrations of glutamine, glutamic acid, alanine, and 2-oxoglutarate in *P. sabinae* T27 WT, and the Δ*ald1* mutant were characterized using UHPLC–HRMS/MS. 50 mL cultures were washed with cold PBS buffer, after which the cultures were harvested and weighed and then rapidly frozen under liquid nitrogen. Five independent samples were analyzed for each condition.

The samples were added to 1 mL 80% methanol, and the mixture was processed by five cycles of 1min ultrasonication with 1min intervals in an ice-water bath. Then, the mixture was incubated for 30 min at −40 °C and 10 min at 4 °C. After centrifugation at 15,000 g and 4 °C for 15 min, the supernatant was evaporated to dryness under nitrogen and reconstituted in 50 μL 50% acetonitrile (including 5μg/mL succinic acid-^13^C_4_, alanine-^13^C_5_-^15^N, and glutamine-^13^C_5_ as internal standards) before performing UHPLC-HRMS/MS analysis. The quality control (QC) sample was obtained by isometrically pooling all the prepared samples.

Chromatographic separation was performed on a ThermoFisher Ultimate 3000 UHPLC system with a Waters BEH Amide column (2.1 mM × 100 mm, 1.7 μm). The injection volume was 2 μL, and the flow rate was 0.25 mL/min. The mobile phases consisted of water with 15 mM ammonium acetate (phase A) and 90% acetonitrile (phase B). Linear gradient elution was performed with the following program: 0 min, 95% B; 4 min, 80% B; 7.5 min, 50% B and held to 9.1 min; 9.1 min, 95% B and held to 10 min. The eluents were analyzed on ThermoFisher Q Exactive™ Hybrid Quadrupole-Orbitrap™ Mass Spectrometry (QE) in Heated Electrospray Ionization Negative (HESI−) mode, separately. The spray voltage was set to 4,000 V. Capillary and probe heater temperatures were separately 320 °C and 320 °C. The sheath gas flow rate was 35 (Arb, arbitrary unit), and the Aux gas flow rate was 10 (Arb). The S-Lens RF level was 50 (Arb). The full scan was operated at a high resolution of 70,000 FWHM (m/z = 200) at a range of 70 to 1,050 m/z with the AGC target set at 3 × 10^6^.

The raw data were processed by Agilent MassHunter Workstation Software (version B.08.00) by using the default parameters and assisting manual inspection to ensure the qualitative and quantitative accuracies of each compound. The peak areas of target compounds were integrated and output for quantitative calculation.

### Expression and Purification of ADH1, AdeR, GS, and GlnR Proteins in *E. Coli*.

The coding regions of *ald1* encoding ADH1, *adeR* encoding AdeR, *glnA* encoding GS, and *glnR* encoding GlnR were PCR amplified from the genomic DNA of *P. sabinae* T27, respectively. These PCR products were cloned into pET-28b to introduce His tags at the N terminus of ADH1, AdeR, GS, and GlnR, respectively, and were then transformed into *E. coli* BL21(DE3). The recombinant *E. coli* strains were cultivated at 37 °C in LB broth supplemented with 50 μg/mL kanamycin until the mid-log phase, when 0.2 mM isopropyl-β-D-thiogalactopyranoside (IPTG) was added, and incubation continued at 16 °C for 8 h. Cells were collected and disrupted in the lysis buffer (50 mM NaH_2_PO_4_, 300 mM NaCl, and 10 mM imidazole) by sonication on ice. Recombinant proteins N-His_6_-ADH1, N-His_6_-AdeR, N-His_6_-GS, and N-His_6_-GlnR in the supernatant were purified on Ni_2_-nitrilotriacetic acid (NTA) resin (Qiagen, Germany) according to the manufacturer’s protocol. Fractions eluted with 250 mM imidazole were dialyzed into binding buffer [20 mM HEPES, pH 7.6, 1 mM EDTA, 10 mM (NH_4_)_2_SO_4_, 1 mM dithiothreitol (DTT), 0.2% Tween 20, and 30 mM KCl) to determine *Km* values.

### Affinity of ADH for Ammonium and Inhibition of GS by Alanine.

The effect of pH on recombinant ADH was determined by using a buffer of 0.1 M citrate-disodium phosphate (pH 3.0 to 8.0) and 0.1 M Tris-HCl (pH 9.0 to 10.0) with pyruvate as the substrate

at 37 °C. To determine the optimum temperature, the reaction mixture was incubated at pH 7.0 for 10 min with pyruvate as the substrate at different temperatures (10 °C ~ 70 °C). The substrate kinetic parameters of NH_4_^+^ were measured under different pH conditions. The *Km* was determined by plotting initial velocity vs. substrate concentration (Michaelis–Menten plot). The data were analyzed using GraphPad Prism 7.0. GS activity was measured as described above. Different concentrations of alanine and NH_4_^+^ were added to test their effects on GS activity. Triplicate measurements were conducted for all the assays.

## Supplementary Material

Appendix 01 (PDF)

## Data Availability

All data arising from this study are included in the main text and *SI Appendix*, with the exception of the raw reads from the RNA-seq analysis, which have been deposited in the NCBI Bioproject PRJNA861244 (65). All research materials supporting this study are available from the corresponding authors.
