# Supplementary material for: Alanine synthesized by alanine dehydrogenase enables ammonium-tolerant nitrogen fixation in Paenibacillus sabinae T27
Source: Proc Natl Acad Sci U S A. 2022 Dec 2;119(49):e2215855119. doi: 10.1073/pnas.2215855119 (PMC9894248; doi:10.1073/pnas.2215855119)
Supplement: Supplementary file 1 — Appendix 01 (PDF) [file pnas.2215855119.sapp.pdf]

Supplementary Appendix for

Alanine synthesized by alanine dehydrogenase enables ammonium tolerant nitrogen fixation in *Paenibacillus sabinae* T27

Qin Li[1], Haowei Zhang[1], Yi Song[1], Minyang Wang[1], Chongchong Hua[1], Yasi Li[1], Sanfeng Chen[1*], Ray Dixon[2*] and Jilun Li[1]

[1]State Key Laboratory for Agrobiotechnology and College of Biological Sciences, China Agricultural University, Beijing, P. R. China.

[2] Department of Molecular Microbiology, John Innes Centre, Norwich, NR4 7UH United Kingdom.

*Corresponding authors: Sanfeng Chen; Ray Dixon
E-mail: chensf@cau.edu.cn (SC); ray.dixon@jic.ac.uk (RD)

This file includes:
Supplementary Materials and methods
Supplementary Figures S1-S8.
Supplementary Tables S1-S5.

Supplementary Materials and Methods

Construction of strains carrying mutations in the GlnR-binding site(s) in the *nif* promoter

Mutations in the GlnR-binding sites were obtained via homologous recombination. A 642 bp *nif* promoter fragment containing both GlnR-binding sites I and II was used as a target for synthesis of variant sequences. PnifR1 contains the mutated GlnR-binding site I where the last six nucleotides 5′-TGACGT-3′ within the 19-bp consensus sequence (GTGTAATGTAATC TGACAT) of GlnR-binding site I were replaced by the restriction site *Kpn*I (GGTACC). PnifR2 carries the mutated GlnR-binding site II where the last six nucleotides 5′-ATAACG-3′ within the 19-bp consensus sequence (GAGTCAGGTTAAC TGACAC) was replaced by the restriction site *Cla*I (ATCGAT). PnifR3 contains both mutant site I and site II where the consensus motifs TGACAT and TGACAC were simultaneously replaced with restriction sites *Kpn*I and *Cla*I respectively. The three synthetic DNA fragments PnifR1, PnifR2 and PnifR3 were then individually cloned into plasmid pUC19 and PCR amplified from the recombinant plasmids. Two homologous arms (flanking the 642 bp region in the *nif* promoter were amplified from the genomic DNA of *P. sabinae* T27 using the primers MRnif1/MRnif2 and primers MRnif5/MRnif6 (Table S5), respectively. Each of the two arms contains a ca. 20 bp overlap with the above described 642 bp DNA fragments (PnifR1, PnifR2 and PnifR3). The two homologous arms and the DNA fragments PnifR1, PnifR2 and PnifR3 were then inserted into the *BamH*I digested plasmid vector pRN5101, yielding the recombinant plasmids pMR1, pMR2, pMR3. Each of these recombinant plasmids was introduced into *P. sabinae* T27 by transformation. The single-crossover transformants were selected for erythromycin resistance (Em^r). Subsequently, the double-crossover transformants were selected from the initial Erythromycin resistance transformants after several rounds of nonselective growth at 39°C. These mutants were confirmed by PCR amplification using the above primers and subsequent digestion with *Kpn*I or *Cla*I, followed by DNA sequencing.

Construction of strains carrying mutations in the AdeR-binding site in the *ald1* promoter

The final six nucleotides of the consensus sequence 5′-CTCATT-3′ in the putative AdeR DNA binding site were replaced by the restriction site *BamH*I (GGATCC) by primer design. The upstream fragment (~1 kb) and downstream fragment (~1 kb) containing the mutated AdeR-binding site were amplified by PCR from genomic DNA. The two fragments were then fused to the *BamH*I digested

pRN5101 vector using Gibson assembly master mix, generating the recombinant plasmid pMAdeR. After transformation into *P. sabinae* T27, single-crossover transformants were selected for erythromycin resistance (Em^r). Subsequently, the double-crossover transformants were selected from the initial erythromycin resistance transformants after several rounds of nonselective growth at 39°C. PCR amplification and subsequent digestion with *BamH*I were used to confirm the mutants. The primers used here are listed in Table S5.

Transcription start site identification

The 5´-RACE method was used to determine the transcription start site (TSS) using the SMARTer RACE cDNA Amplification Kit (Clontech). Gene-specific primers are listed in Table S5. The PCR product was cloned into the pMD18-T vector and then sequenced.

Electrophoretic mobility shift assays (EMSAs)

EMSAs were performed as described previously using a DIG Gel Shift Kit (2nd Generation; Roche, USA) (1). Promoter fragments from the *nif* operon were synthesized by Sangon Biotech Co., Ltd (Shanghai) corresponding to the sequence of the top strand and the complementary DNA strand. The two strands were annealed and then labeled at the 3´ end with digoxigenin (DIG) using terminal transferase, and used as probes in EMSAs. Each binding reaction (20 µl) consisted of 1 µg poly [d(A-T)], 0.3 nM labelled probe, and various concentrations of purified His6-GlnR in the binding buffer. Reaction mixtures were incubated for 30 min at 25˚C, analyzed by electrophoresis using a native 5% polyacrylamide gel run at 4˚C with 0.5×TBE as running buffer, and electrophoretically transferred to a positively charged nylon membrane (GE healthcare, UK). Labelled DNAs were detected by chemiluminescence according to the manufacturer's instructions and recorded on X-ray film. The primers used here are listed in Table S5.

Surface plasmon resonance (SPR) detection

SPR experiments (2) were carried out using Biacore 3000 SPR sensor (Biacore AB, Uppsala, Sweden). All assays were carried out at 25˚C. HBS buffer supplied with 3 mM $MgCl_2$ (10 mM HEPES pH7.4, 300 mM NaCl, 3 mM $MgCl_2$-$6H_2O$, 0.2 mM EDTA, and 0.005% Tween-20) was used as the running buffer.

Protein-DNA interaction assays were performed with Sensor Chip SA. First, a biotinylated single-stranded DNA capture linker (biotin GCAGGAGGACGTAGGGTAGG) was irreversibly bound to the chip. DNA oligomer used for SPR assays (Table S4) were designed and synthesized based on the *nif* promoter region harboring GlnR-binding sites and containing a single-stranded overhang complementary to the linker. Then a partially double-stranded DNA oligomer that contained either GlnR-binding site I or site II in the double-stranded region with a single-stranded overhang complementary to the capture linker was bound to the chip, reaching a signal of 250 RU. Control DNA was bound onto flow cell 1 (FC1), and DNA fragments containing GlnR binding sites were bound to flow cells 2 and 3 (FC2, FC3). GlnR with or without 25 nM GS protein was injected at a flow rate of 30 ul/min.

Reference

1. L. Wang, et al., A minimal nitrogen fixation gene cluster from *Paenibacillus* sp. WLY78 enables expression of active nitrogenase in *Escherichia coli*. *PLoS Genet.* **9**, e1003865 (2013).
2. CE. Stevenson, et al. Investigation of DNA sequence recognition by a streptomycete MarR family transcriptional regulator through surface plasmon resonance and X-ray crystallography. *Nucleic Acids Res*. 41: 7009–222 (2013).

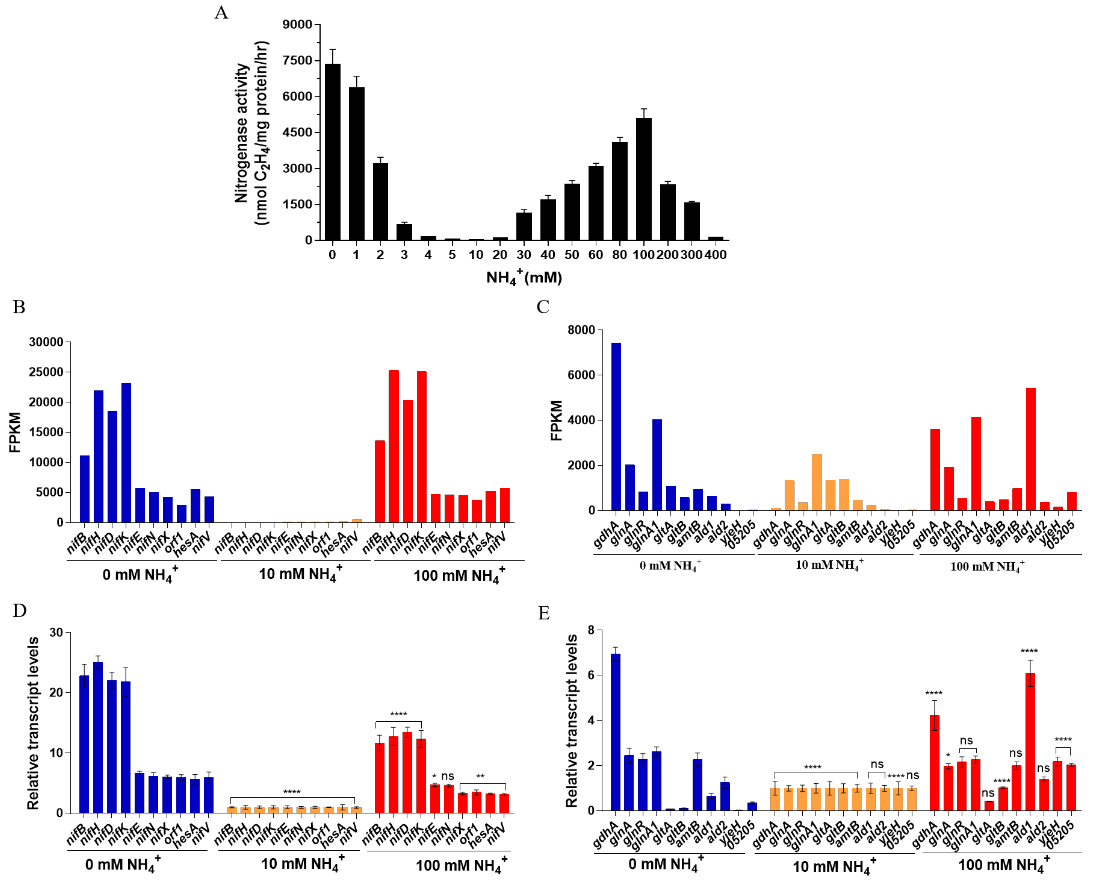

Fig. S1. Nitrogenase activity and transcription analysis analysis of *P. sabinae* T27 grown with different concentrations of NH_4^+. (A) Nitrogenase activities of *P. sabinae* T27 grown in medium containing 0-400 mM NH_4^+. (B) Expression levels of *P. sabinae* T27 *nif* genes when grown with the indicated concentrations of NH_4^+. (C). Expression levels of genes involved in ammonia assimilation in *P. sabinae* T27 when grown with ammonium concentrations indicated in the legend. FPKM (Fragments Per Kilobase of transcript per Million reads mapped) was used for comparative analysis of gene expression levels. (D) qRT-PCR analysis of the relative transcript levels of the *nif* genes. (E) qRT-PCR analysis of the relative transcript levels of nitrogen metabolism and associated genes. Bars represent mean ± standard deviation (n = 3). Statistical analysis was carried out by two-way ANOVA with Tukey's multiple comparisons used to compare means. Not significant (ns P > 0.05), *P < 0.05, **P < 0.01, ***P < 0.001, ****P < 0.0001. Bacteria grown without ammonium (0 mM NH_4^+) were used as the reference group for statistical comparisons.

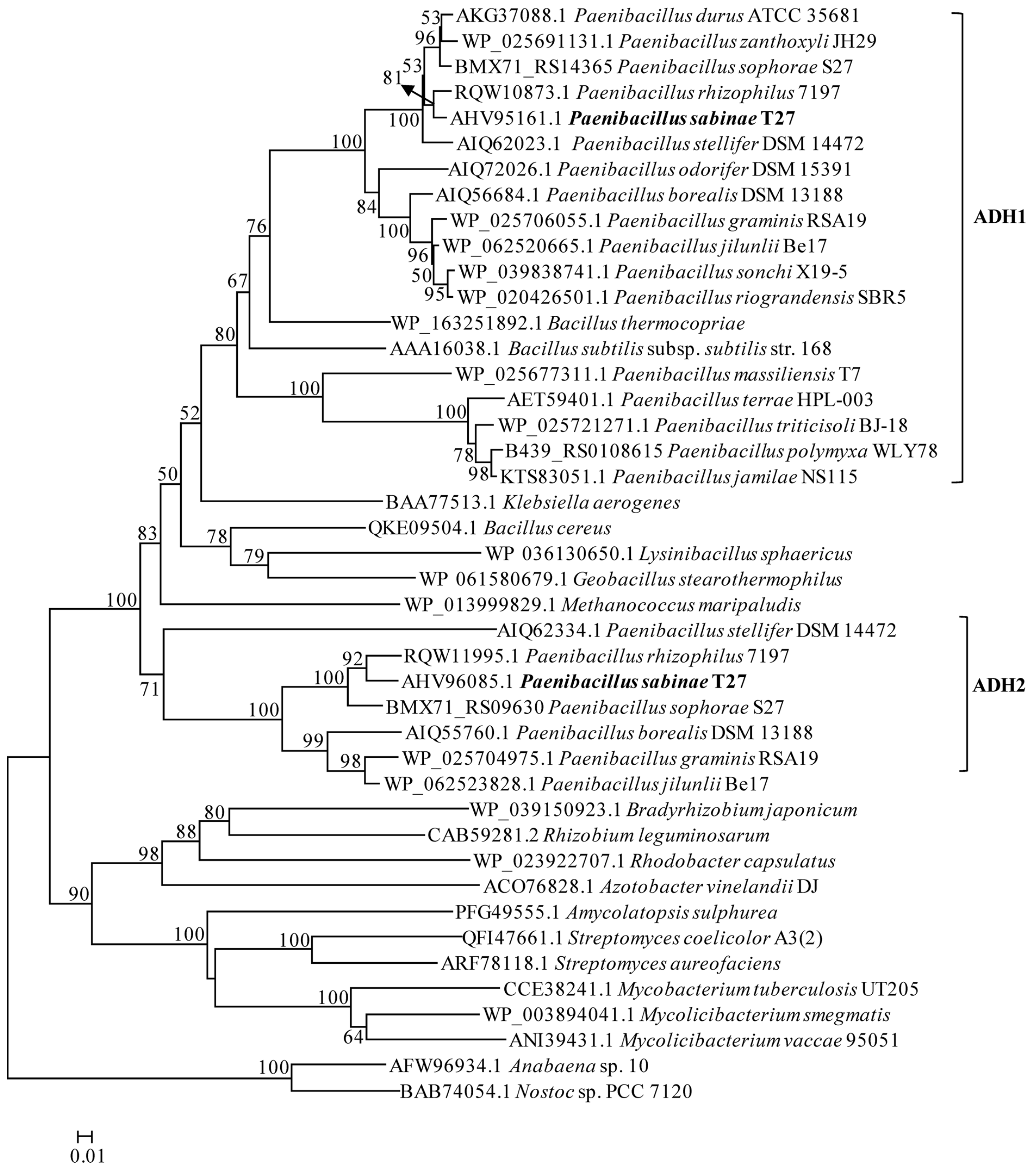

Fig. S2. Maximum likelihood phylogenetic tree of ADH (alanine dehydrogenase) proteins from *Paenibacillus sabinae* T27 and other bacteria

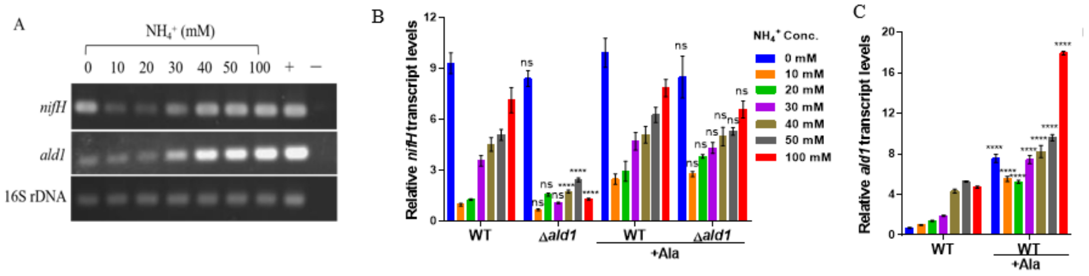

Fig. S3. Analysis of *nifH* and *ald* transcripts in response to alanine addition in *P. sabinae* T27. (A) RT-PCR analysis of *ald1* and *nifH* transcripts cultivated in minimal medium containing the ammonium concentrations indicated at the top of the figure. Lanes labeled "+" indicate positive controls in which genomic DNA was used as template in the RT-PCR. Lanes labeled "-" indicate negative controls in which no reverse transcriptase was added to the RT-PCR reaction**.** (B) qRT-PCR analysis of *nifH* expression in the wild-type (WT) and Δ*ald1* mutant in the presence or absence of 5 mM alanine, in response to the ammonium concentrations indicated in the colored legend. (C) qRT-PCR analysis of *ald1* expression in the presence or absence of 5 mM alanine in response to the ammonium concentrations indicated. Bars represent mean ± standard deviation (n = 3). Statistical analysis in panels B and C was carried out by two-way ANOVA with Tukey's multiple comparisons used to compare means. Not significant (ns $P > 0.05$), ****$P < 0.0001$. The wild type and Δ*ald1* strains grown in the absence of alanine were used as reference groups for statistical comparisons.

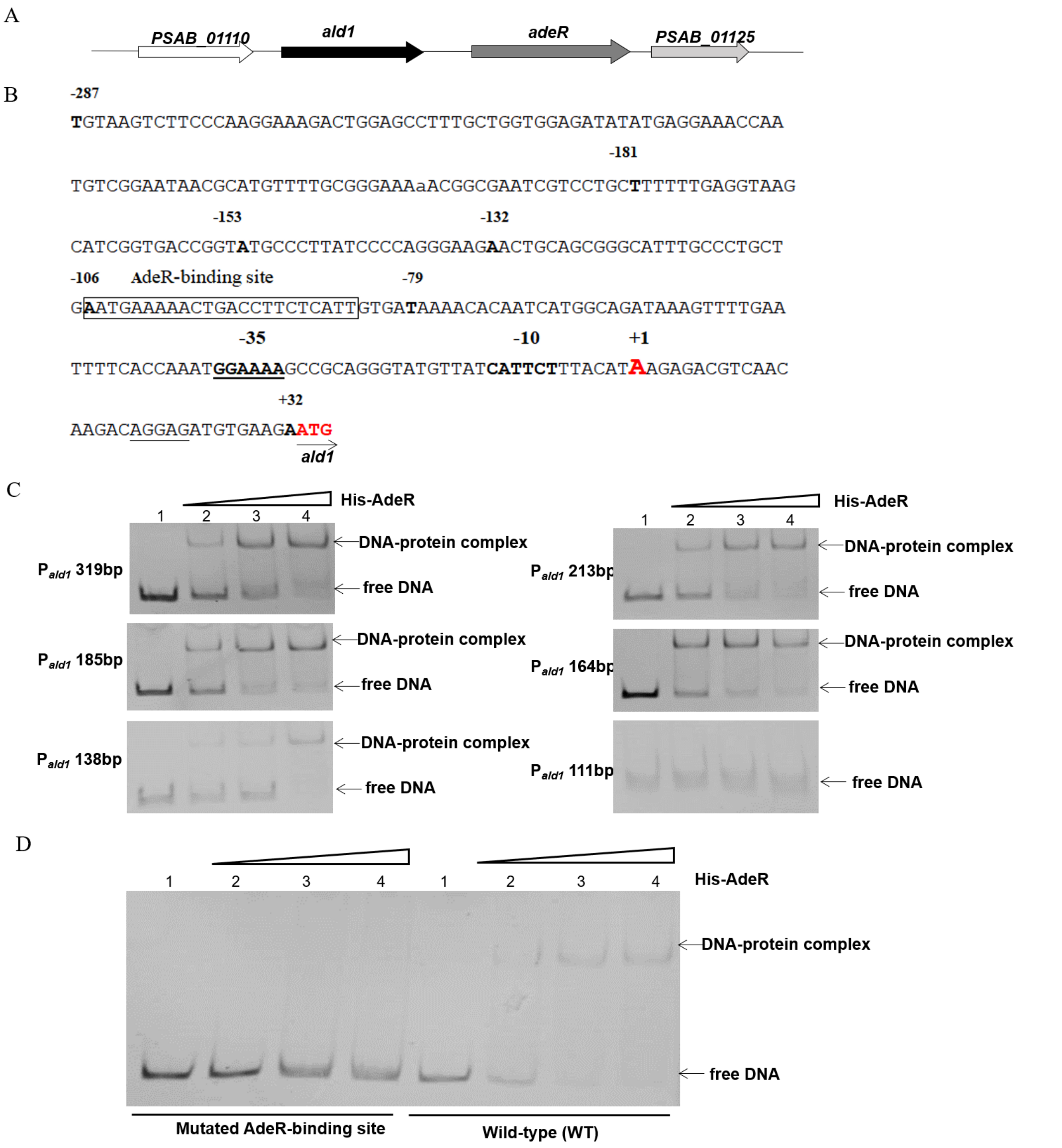

Fig. S4. Genetic organization of *ald1* and *adeR* and the characteristics of the *ald1* promoter in *P. sabinae* T27. (A) Organization of the *ald1* and *adeR* genes in *P. sabinae* T27. (B) Transcription start site (+1) of the *ald1* gene determined by 5´ RACE. Nucleotide positions are numbered relative to the transcriptional start site (TSS) of *ald1*. Putative -10 and -35 sequences and RBS (ribosome binding site) are also shown. The putative AdeR binding site is boxed. (C) EMSA experiments showing the binding of purified AdeR to different promoter regions containing AdeR-binding sites. The various promoter DNA templates indicated in the figure were incubated with increasing concentrations of purified AdeR. Lane 1 contained no AdeR. Lanes 2–4 contained 100 nM, 300 nM and 600 nM His-tagged AdeR, respectively. (D) EMSA showing no binding of the purified AdeR to the mutated GlnR-binding site, with the 40-bp DNA fragment containing the wild-type AdeR-binding site as a control. Lane 1 contained no AdeR. Lanes 2–4 contained increasing concentrations of His-tagged AdeR (100, 300 and 600 nM).

.

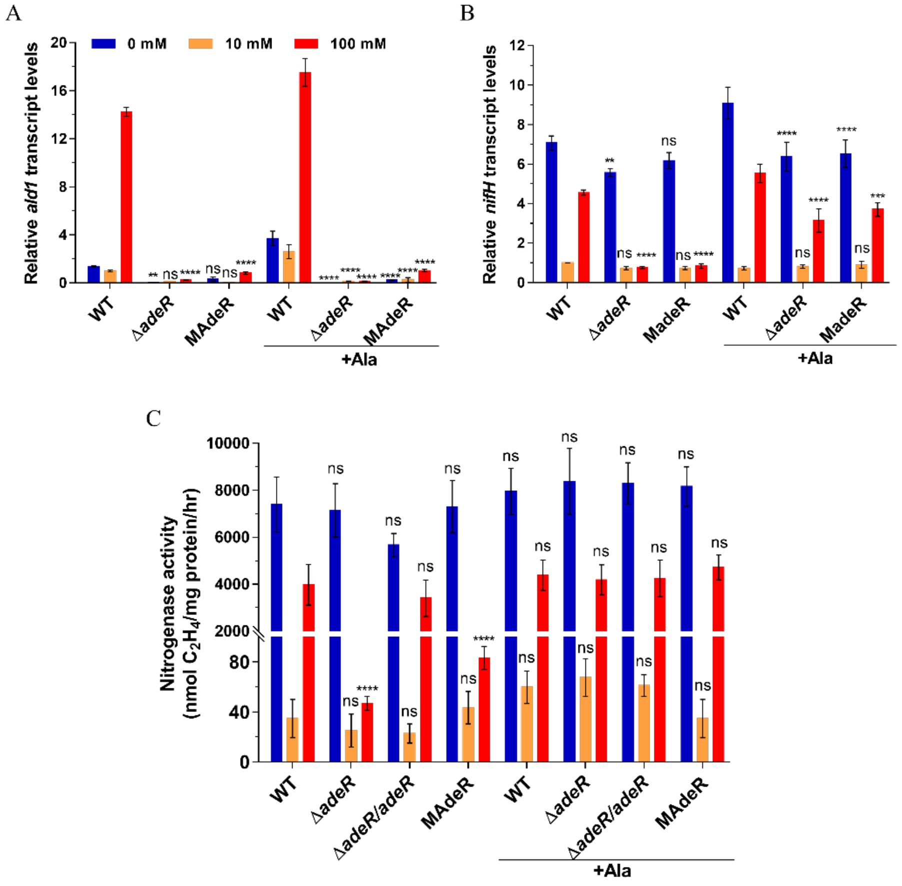

Fig. S5. Influence of AdeR-mediated gene regulation on *ald1* and *nif* gene expression. (A) Relative transcript levels of *ald1* in WT (wild-type), Δ*adeR* and MAdeR (mutated AdeR-binding site) mutants in absence or presence of 5 mM alanine was determined by qRT-PCR. (B) Relative *nifH* transcript levels in WT (wild-type), Δ*adeR* and MAdeR mutants in absence or presence of 5 mM alanine as determined by qRT-PCR. Relative qRT-PCR transcript levels were arbitrarily set to 1.0 in the WT (wild-type) strain grown in the presence 10 mM NH_4^+ in the absence of L-alanine. (C) Nitrogenase activities in WT (wild-type), Δ*adeR* and MAdeR mutants and complemented strain Δ*adeR*/*adeR*. Bars represent mean ± standard deviation (n = 3). Statistical analysis was carried out by two-way ANOVA with Tukey's multiple comparisons used to compare means. The wild type strain was used as reference group for statistical comparisons. Not significant (ns P > 0.05), ***P < 0.001, ****P < 0.0001.

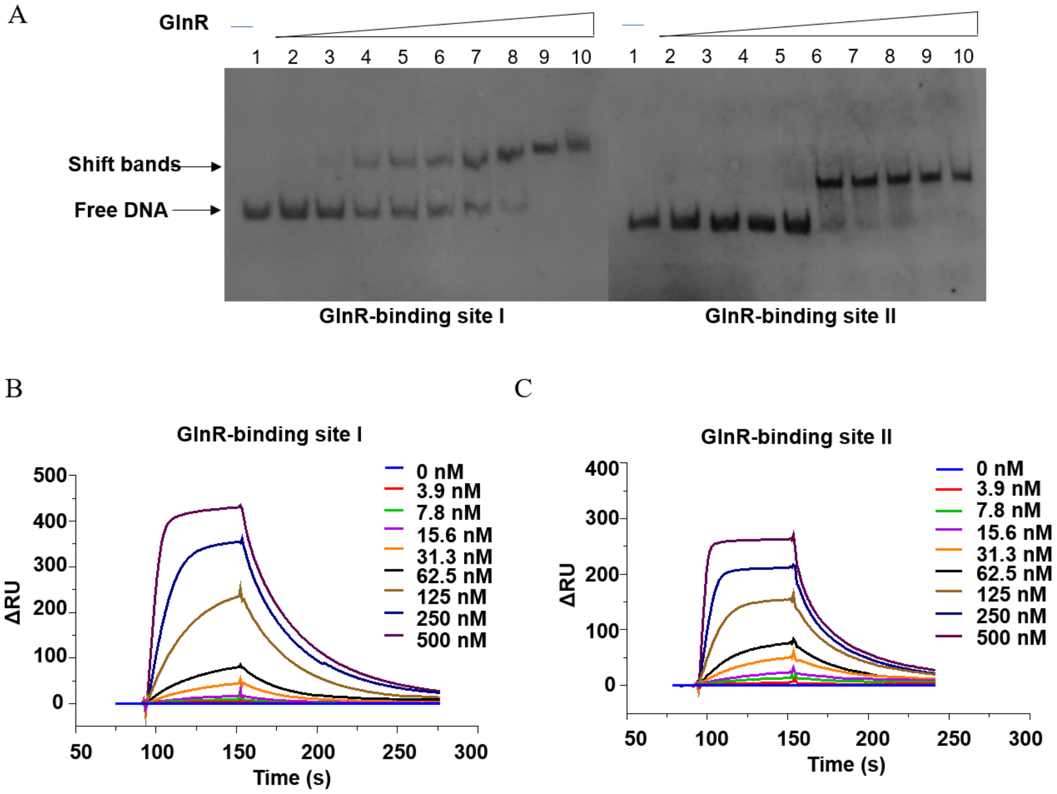

D

	K_A (nM)	K_D (nM)
**GlnR-binding site I**	**6.02×10^{6}**	**1.66×10^{-7}**
**GlnR-binding site II**	**1.16×10^{7}**	**8.62×10^{-8}**

Fig. S6. Binding affinity of GlnR to the two GlnR binding sites in the *nif* promoter region of *P. sabinae* T27. (A) EMSA showing the *in vitro* binding of GlnR to the two GlnR-binding sites. Two DNA fragments: a 43 bp DNA fragment harboring GlnR-binding site I and a 41 bp DNA fragment carrying GlnR-binding site II, were synthesized and biotin-labeled. The biotin-labeled DNA fragments were incubated with His-GlnR. Lane 1 contained no GlnR. Lanes 2–10 contained increasing concentrations of His-tagged GlnR (4, 8, 16, 32, 64, 128, 256, 512, 1024 nM). (B) Surface plasmon resonance (SPR) analysis of GlnR binding to site I. (C) SPR assay of GlnR binding to site II. (D) Binding affinity of GlnR to site I and site II. The concentration of GlnR was increased from 0 nM to 500 nM as indicated in the legend.

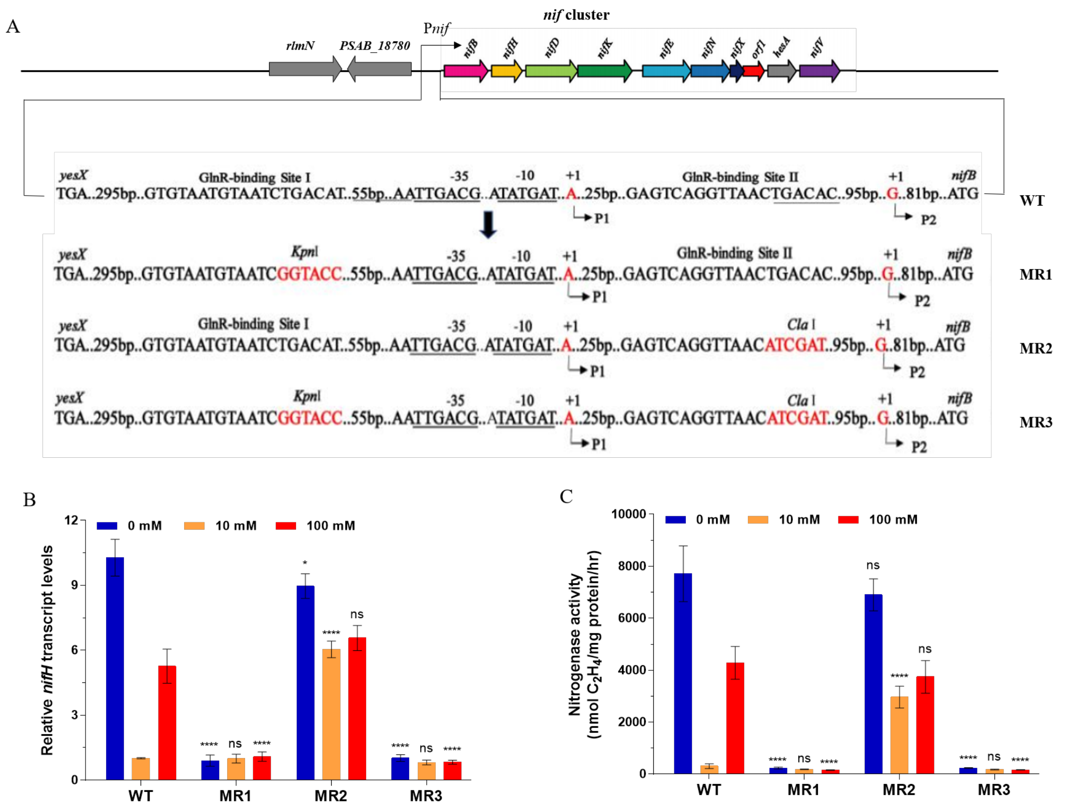

Fig. S7. Mutation of the GlnR-binding sites in the *nif* promoter region and analysis of nitrogenase activity and *nif* gene transcription in mutants carrying altered GlnR-binding sites. (A) Diagram showing the *nif* gene cluster and site-specific mutagenesis of the GlnR-binding sites in the *nifB* promoter. (B) qRT-PCR analysis of the relative transcript levels of the *nifH* gene in the wild-type (WT) and mutant strains grown in minimal medium containing 0 mM, 10 mM and 100 mM NH_4^+respectively. The transcript levels of *nifH* in the WT strain grown with 10 mM NH_4^+ was arbitrarily set to 1.0. Results are representative of at least three independent experiments. (C) Nitrogenase activity in wild-type (WT) and three mutants MR1, MR2 and MR3 grown in minimal medium containing 0 mM, 10 mM and 100 mM NH_4^+, respectively. Error bars indicate SD. Not significant (ns $P > 0.05$), $*P < 0.05$, $****P < 0.0001$. WT bacteria were used as a reference group for statistical comparisons.

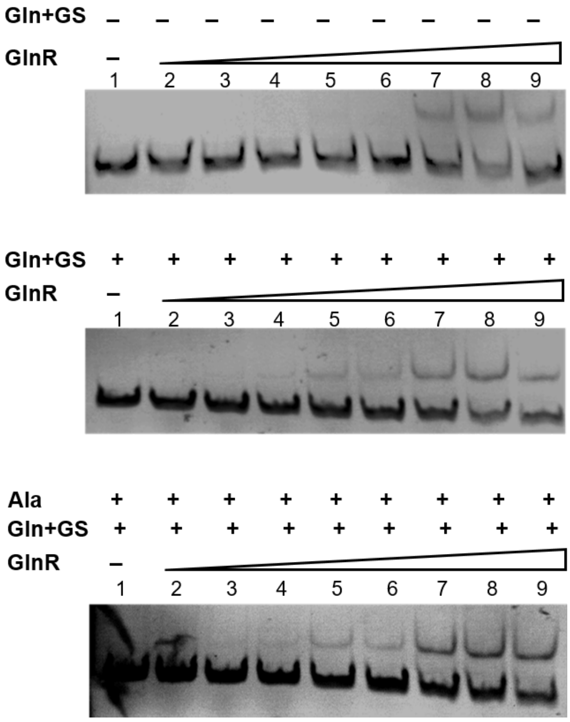

Fig. S8. Influence of FBI-GS and alanine on the binding affinity of GlnR to binding II site in the *nif* promoter region of *P. sabinae* T27. EMSA experiments were carried out with a biotin-labeled 41 bp DNA fragment carrying GlnR-binding site II. This fragment was incubated with different concentration of His-GlnR supplemented with or without FBI-GS (5 mM glutamine and 500 nM His-GS) or with mixture of FBI-GS and Ala (5 mM). Plus and minus signs indicate the presence or absence of reaction components respectively (e.g. Lane 1 contained no GlnR). Lanes 2–9 contained increasing concentrations of His-tagged GlnR (2, 4, 8, 16, 32, 64, 128, 256 nM).

Table S1. Expression levels of the genes involved in nitrogen fixation and ammonia assimilation in *P. sabinae* T27 grown with three different concentrations of NH_4^+ (0 mM, 10 mM and 100 mM)

Locus tag	Gene name	Gene product	FPKM[a] T27-0[b]	FPKM T27-10[c]	FPKM T27-100[d]	T27-0/T27-10[e] log2 FC[h]	T27-0/T27-10[e] p-value	T27-100/T27-0[f] log2FC	T27-100/T27-0[f] p-value	T27-100/T27-10[g] log2FC	T27-100/T27-10[g] p-value
PSAB_18775	*nifB*	Nitrogenase cofactor biosynthesis protein	13580.511	11.417	11100.551	10.2	1.16E-25	-0.29	8.14E-01	9.91	7.70E-26
PSAB_18770	*nifH*	nitrogenase iron protein subunit	25316.718	17.599	21876.26	10.48	1.95E-26	-0.21	7.23E-01	10.27	7.19E-27
PSAB_18765	*nifD*	Nitrogenase molybdenum-iron protein alpha chains	20339.415	8.35	18564.778	11.23	1.04E-28	-0.13	6.32E-01	11.1	2.46E-29
PSAB_18760	*nifk*	Nitrogenase molybdenum-iron protein beta chains	25117.187	8.708	23088.684	11.48	1.92E-29	-0.12	6.21E-01	11.36	3.73E-30
PSAB_18755	*nifE*	nitrogenase MoFe cofactor biosynthesis protein	4696.231	99.046	5709.53	5.57	4.06E-12	0.28	2.69E-01	5.85	6.08E-14
PSAB_18750	*nifN*	nitrogenase molybdenum-cofactor biosynthesis protein	4606.468	96.55	4963.752	5.57	3.80E-12	0.11	4.00E-01	5.68	1.87E-13
PSAB_18745	*nifX*	nitrogen fixation protein	4536.004	81.766	4164.944	5.79	1.29E-12	-0.12	6.39E-01	5.67	3.00E-13
PSAB_18740	*orf1*	hypothetical protein PSAB_18740	3701.501	69.778	2889.134	5.73	2.03E-12	-0.36	9.08E-01	5.37	2.22E-12
PSAB_18735	*hesA*	UBA/THIF-type NAD/FAD binding protein	5229.197	167.528	5477.993	4.96	2.11E-10	0.07	4.38E-01	5.03	1.44E-11
PSAB_18730	*nifV*	trans-homoaconitate synthase	5747.651	441.464	4321.476	3.7	5.55E-07	-0.41	9.61E-01	3.29	8.31E-07
PSAB_12720	*gdhA*	Glutamate dehydrogenase	7432.62	105.28	3599.21	6.14	8.65E-14	-1.05	3.59E-01	5.09	9.40E-12
PSAB_14080	*glnA*	Glutamine synthase	2020.35	1325.32	1928.87	0.61	4.19E-01	-0.07	5.64E-01	0.54	2.20E-01
PSAB_14085	*glnR*	GlnR regulator	839.26	347.11	546.45	1.27	6.87E-02	-0.62	7.79E-01	0.65	1.63E-01
PSAB_08210	*glnA1*	Glutamine synthase	4044.46	2472.5	4140.73	0.71	3.36E-01	0.03	4.65E-01	0.74	1.26E-01
PSAB_04930	*gltA*	Glutamate synthase large subunit	1076.9	1330.56	388.62	-0.31	5.54E-01	-1.47	1.21E-01	-1.78	2.36E-02
PSAB_01395	*gltB*	Glutamate synthase small subunit	574.77	1390.06	489.28	-1.27	4.21E-02	-0.23	7.46E-01	-1.51	6.16E-02
PSAB_09785	*amtB*	Ammonium transporter	940.96	459.76	979.99	-1.03	1.48E-01	0.06	4.43E-01	1.09	4.14E-02
PSAB_01115	*ald1*	Alanine dehydrogenase	627.46	217.94	5415.16	1.53	3.03E-02	3.11	8.54E-07	4.63	1.81E-10
PSAB_05745	*ald2*	Alanine dehydrogenase	287.793	40.393	384.877	2.83	9.35E-05	0.42	1.92E-01	3.25	1.27E-06
PSAB_13530	*yjeH*	Amino acid transporter	3.24	5.07	152.31	-0.63	3.73E-01	5.51	2.45E-12	4.88	1.34E-10
PSAB_05205	PSAB_05205	Drug/metabolite transporter permease	6.96	13.09	805.05	-0.9	1.97E-01	6.83	1.32E-16	5.93	7.09E-14

[a]FPKM (Fragments Per Kilobase of transcript per Million reads mapped) was used for comparative analysis of gene expression levels.

[b]T27-0 represents the transcriptional profile of *P. sabinae* T27 grown in 0 mM NH_4^+.

[c]T27-10 represents the transcriptional profile of *P. sabinae* T27 grown in 10 mM NH_4^+.

[d]T27-100 represents the transcriptional profile of *P. sabinae* T27 grown in 100 mM NH_4^+.

[e]T27-0/T27-10 indicates the differences between T27-0 versus T27-10

[f]T27-100/T27-0 indicates the differences between T27-100 versus T27-0

[g]T27-100/T27-10 indicates the differences between T27-100 versus T27-10

[h]log2 FC indicates $\log_2$ (Fold Change).

Table S2. Intracellular concentrations of metabolites in wild-type and the *ald1* mutant of *P. sabinae* T27 grown with different concentrations of ammonia

Strain	NH_4^+ Treatment	metabolite concentration (mM) Glutamine	Glutamate	Alanine	2-oxoglutarate	Gln/2-OG	Glu/Gln
WT	0 mM	0.060	2.636	0.762	0.025	2.399	43.889
	10 mM	0.522	7.646	4.143	0.020	26.443	14.750
	100 mM	0.109	4.449	14.067	0.030	3.601	41.126
Δ*ald1*	0 mM	0.044	2.940	0.929	0.038	1.137	67.995
	10 mM	0.499	5.197	0.620	0.019	26.390	10.483
	100 mM	0.317	5.599	1.838	0.013	24.012	17.796
Δ*ald1*+Ala[a]	0 mM	0.085	3.492	5.487	0.033	2.557	41.571
	10 mM	0.337	2.549	3.916	0.026	13.003	7.619
	100 mM	0.146	4.715	7.260	0.033	4.387	32.492

[a] L-alanine was added to the medium (5 mM, final concentration)

Table S3. Enzyme activities in *P. sabinae* T27 wild-type (WT) and the Δ*ald1* mutant grown with different concentration of NH_4^+

Strain	NH_4^+ treatment	ADH	GDH	GS	GOGAT
		nmol·min^{-1}·mg protein^{-1}			
WT	0 mM	544.32	2708.71	3.24	187.58
	10 mM	299.65	1470.04	0.73	402.02
	100 mM	3110.22	1781.86	1.78	290.80
Δ*ald1*	0 mM	412.47	1130.61	2.22	93.73
	10 mM	215.73	1023.54	0.78	175.46
	100 mM	148.38	1213.60	1.29	208.49

Table S4. Bacterial strains and plasmids used in this study

Strain /plasmid	Relevant characteristics	Source or reference
Paenibacillus sabinae		
T27	Wild-type strain	Laboratory stock
Δ*ald1*	*ald1* in-frame deletion mutant of *P. sabinae* T27	This study
Δ*ald2*	*ald2* in-frame deletion mutant of *P. sabinae* T27	This study
Δ*yjeH*	*yjeH* in-frame deletion mutant of *P. sabinae* T27	This study
Δ*PSAB_05205*	*PSAB_05205*in-frame deletion mutant of *P. sabinae* T27	This study
Δ*adeR*	*adeR* in-frame deletion mutant of *P. sabinae* T27	This study
Δ*ald1ald2*	double *ald* deletion mutant strains of *P. sabinae* T27	This study
Δ*ald1/ald1*	Complementation strain of *P. sabinae* T27 Δ*ald1* with *P. sabinae* T27 *ald1* carried in plasmid pCald1	This study
Δ*adeR/adeR*	Complementation strain of *P. sabinae* T27 Δ*nifB1* with *P. sabinae* T27 *nifB1* carried in plasmid pCadeR	This study
MAdeR	A derivative of *P. sabinae* T27 with site-specific mutation at AdeR-binding site	This study
MR1	A derivative of *P. sabinae* T27 with site-specific mutation at GlnR-binding site I	This study
MR2	A derivative of *P. sabinae* T27 with site-specific mutation at GlnR-binding site II	This study
MR3	A derivative of *P. sabinae* T27 with site-specific mutations at both GlnR-binding sites	This study
Escherichia coli		
JM109	General cloning host*; recA1, endA1, gyrA96, thi-1, hsdR17, supE44, relA1, Δ(lac-proAB)*/F'[*traD36, proAB+, lacIq, lacZ*ΔM15]	Sangon Biotech Co.
BL21 (DE3)	Host for protein overexpression; F^{-}, *ompT, gal, dcm, lon, hsdSB*($r_B^- m_B^-$-), λ (DE3 [*lacI, lacUV5-T7 gene 1, ind1, sam7, nin5*])	Sangon Biotech Co.
**Plasmids**		
pRN5101	Temperature-sensitive *E. coli-Bacillus* shuttle vector, $Amp^R Em^R$	(40)
pET-28b	Vector for His_6-tagged protein overexpression in *E. coli*, T7 promoter and T7 terminator, pBR322 origin, Kan^R	Novagen
pDald1	Δ*ald1* deletion vector based on pRN5101	This study
pDald2	Δ*ald2* deletion vector based on pRN5101	This study
pDyjeH	Δ*yjeH* deletion vector based on pRN5101	This study
pDPSAB_05205	Δ*PSAB_05205* deletion vector based on pRN5101	This study
pDadeR	Δ*adeR* deletion vector based on pRN5101	This study
pCald1	Δ*ald1* complemented vector with *ald1* in pRN5101	This study
pCadeR	Δ*ald2* complemented vector with *ald2* in pRN5101	This study
pMAdeR	A derivative of vector pRN5101 for deletion of AdeR-binding site	This study
pMR1	A derivative of vector pRN5101 for mutation of GlnR-binding site I	This study
pMR2	A derivative of vector pRN5101 for mutation of GlnR-binding site II	This study
pMR3	A derivative of vector pRN5101 for mutation of both GlnR-binding sites	This study
pETADH1	*Ald1* overexpression vector based on pET-28b	This study

pETAdeR	*adeR* overexpression vector based on pET-28b	This study
pETGlnR	*glnR* overexpression vector based on pET-28b	This study
pETGlnA	*glnA* overexpression vector based on pET-28b	This study

Table S5. Primers used in this study

Primer	Sequence	Purpose
**For qRT-PCR**		
Q16S-F	TTTGTCGTCAGCCTCGTGTTCGTG	qRT-PCR for control (16S rDNA)
Q16S-R	ATCCCCACCTTCCTCCGGTTTG	
QnifBF	GACTTTGTCTCCGCCTCG	qRT-PCR for *nifB*
QnifBR	AAATCCGTCAGCGTGTTAT	
QnifHF	CGTCTTCTACCACACCGAACT	qRT-PCR for *nifH*
QnifHR	CACAACCAAGCCAACGAATA	
QnifDF	CACAGTCATATCCATGTCCTTGG	qRT-PCR for *nifD*
QnifDR	GTCAAATGCACTCCTGGGATTA	
QnifKF	GCATCGTAATCCGATTATCGG	qRT-PCR for *nifK*
QnifKR	AGCGAACCAAATCAACGAGCC	
QnifEF	TTACGGCACTAATCGGCGAAG	qRT-PCR for *nifE*
QnifER	GTTCACACCAAGCGGAGTCTC	
QnifNF	GGCTCTGAATGCGGTACTGTC	qRT-PCR for *nifN*
QnifNR	GCGTTACGGAGAAGGCAAGG	
QnifXF	ACATTACGAAGAGCAGCAGTGAG	qRT-PCR for *nifX*
QnifXR	CGATCTTGCCGAACTCATCCTG	
Qorf1F	AATCATCAAGCCGCAGTTC	qRT-PCR for *orf1*
Q0rf1R	ATCGGCGGAGGATAAAGC	
QhesAF	GACGGAGCAGCAGCGATATG	qRT-PCR for *hesA*
QhesAR	TCTTGCCTTCATGAGCGAGTATC	
QnifVF	GCGGCGGAATATGCGATAGG	qRT-PCR for *nifV*
QnifVR	GATGATGTGCGGAGACGGTATC	
QgdhAF	CCTCTATCGCTCAGCACTTTC	qRT-PCR for *gdhA*
QgdhAR	AACGGGACTCCTTTGGAAC	
QglnAF	GGTGCCATTTCCTTGATTCT	qRT-PCR for *glnA*
QglnAR	GGCTGATTTCGGGATAAACT	
QglnRF	CGGGCAACCAGAGATTATTC	qRT-PCR for *glnR*
QglnRR	CGGAAACCAACTCCTGCTT	
QglnA1F	TTTCACTCCTGATGGCGA	qRT-PCR for *glnA1*
QglnA1R	CTGTTCATTCCGCTCTCGT	
QgltAF	GCATCGGCGGCAAGAGTAATACC	qRT-PCR for *gltA*
QgltAR	GAATCTCGTCGGCGTTCACCAG	
QgltBF	CCAACCGTACACGAGCCTTCAC	qRT-PCR for *gltB*
QgltBR	GACATGGCTGGCGGCACTTC	
QamtBF	AATGGCGTTTGCTGCTGT	qRT-PCR for *amtB*
QamtBR	ACCCGTCAGATGGACTACTGT	
Qald1F	CGTTACCATCGTTGACCTGA	qRT-PCR for *ald1*
Qald1R	CTTCACCATTTCTTCGGTTACC	
Qald2F	GACAAAGGAGCGGAGATTCT	qRT-PCR for *ald2*

Qald2R	TAACCAACGGCAACAACG	
QyjeHF	TTACGGGTCTGTTCATCTGC	qRT-PCR for *yjeH*
QyjeHR	GAGGGTAGTCAGCGACACAA	
Q05205F	TTATTACACCCAGCAGCGTC	qRT-PCR for *PSAB_05205*
Q05205R	TTTATCTCACCGTCGTCGG	
QadeRF	TTCCTGACCACGGAGCATA	qRT-PCR for *adeR*
QadeRR	ACTCGCTGTAATGGAGGCTG	
**Construction of Δ*ald1*, Δ*ald2*, Δ*yjeH*, Δ*PSAB_05205*, Δ*ald1*Δ*ald2*, Δ*adeR* mutants and complementation strains**		
ald1UF	ACGATGCGTCCGGCGTAGAGGATCCGCAGCAGATTGCGCTGCG	In-frame deletion of *ald1*
ald1UR	CTCTTGGGACGGTACTCCGATGATCATTCT	
ald1DF	TCGGAGTACCGTCCCAAGAGTTTACCCTG	
ald1DR	GCGACCACACCCGTCCTGTGGATCCTAAAATAACCCGAAGGCAG	
yjeHUF	ACGATGCGTCCGGCGTAGAGGATCCATGTTTTTGCACCATTTC	In-frame deletion of *yjeH*
yjeHUR	GACACTTCACCATTACAATCATCCAGCC	
yjeHDF	GATTGTAATGGTGAAGTGTCCTAATAGCAC	
yjeHDR	GCGACCACACCCGTCCTGTGGATCGAGGTACCGATCCCTTTC	
05205UF	ACGATGCGTCCGGCGTAGAGGATCCTCGTCCTCATGCCTTACATT	In-frame deletion of *PSAB_05205*
05205UR	AATGAACATGATGAGGATTAATGAGCGCTA	
05205DF	TAATCCTCATCATGTTCATTGCTCCTTTGC	
05205DR	GCGACCACACCCGTCCTGTGGATCCACACCGGAACGGTATACC	
ald2UF	ACGATGCGTCCGGCGTAGAGGATCCACATTGGGTATCGTTCCG	In-frame deletion of *ald2*
ald2UpR	AGCTTCCAATTTCGGTATGCCGACAATC	
ald2DF	GCATACCGAAATTGGAAGCTTTGAAGGAG	
ald2DR	CGCAAAAGACATAATCGATAAGCTTGAGGCTTTCCCCATACTG	
adeRUF	ACGATGCGTCCGGCGTAGAGGATCCGGCGGCGTTGTCGGAAC	In-frame deletion of *adeR*
adeRUR	ACCTTCCGCAGCTTCGGTCACTGTTCCCAC	
adeRDF	TGACCGAAGCTGCGGAAGGTTGATGGAAG	
adeRDR	GCGACCACACCCGTCCTGTGGATCGGCCCATTGTATAGACC	
Cald1F(BamHI)	ACGATGCGTCCGGCGTAGAGGATCCGTAAGTCTTCCCAAGGAAAG	Complementation of Δ*ald1* with *ald1*gene in vector pRN5101
Cald1R(BamHI)	GCGACCACACCCGTCCTGTGGATCCCAGCCGACTTTTTGTTCTTC	
CadeRF(B	ACGATGCGTCCGGCGTAGAGGATCCGCCATGT	Complementation of

amHI)	CCCAAGAGTTTAC	Δ*adeR* with *adeR* gene
CadeRR(BamHI)	GCGACCACACCCGTCCTGTGGATCCGCTTATCCGCCATATCTTC	in vector pRN5101
**Overexpression of His^6-tagged protein**		
ALd1F（BamHI）	CGCGGATCCAATGATCATCGGAGTACCTAAG	Overexpression of His^6-ADH1 in *E. coli*
ALd1R（SalI）	ACGCGTCGACTTAGATCAGGGTAAACTCTTGG	
AdeRF (BamHI)	CGCGGATCCAGTGACCGAAGCGAGCTTATC	Overexpression of His^6-AdeR in *E. coli*
AdeRR（SalI）	ACGCTCAACCTTCCGCAAACCACT	
GlnAF（EcoRI）	CCGGAATTCAGTGAGCTTTAGTAAAGAAGATA	Overexpression of His^6-GS in *E. coli*
GlnAR（SalI）	ACGCGTCGACAAGGGCTTCCTTAGTATTC	
GlnRF（EcoRI）	CCGGAATTCCATGGGTGATGAAATCCGCAGAA	Overexpression of His^6-GlnR in *E. coli*
GlnRR（salI）	ACGCGTCGACCCTTTCGCGCTTCTGTATGTG	
**EMSA, 5’ RACE and SPR**		
ald1-GSP1	GATTACGCCAAGCTTTTATCCTTCAGCGCCTTCGCCAGAGCA	Identification of transcription start site (TSS) of *ald1* by RACE
nifR1F	AACGGAAACATGATACATATGTGTAATGTAATCTGACATTTGT	EMSA probe of GlnR-binding site I in the *nif* promoter region
nifR1R	ACAAATGTCAGATTACATTACACATATGTATCATGTTTCCGTT	
nifR2F	TTTTCAAGAAAGGTTGGAGTCAGGTTAACTGACACGTCGGATG	EMSA probe of GlnR-binding site II in the *nif* promoter region
nifR2R	CATCCGACGTGTCAGTTAACCTGACTCCAACCTTTCTTGAAAA	
nifR1MF	AACGGAAACATGATACATATGTGTAATGTAATCGGTACCTTGT	EMSA probe of mutated GlnR-binding site I in the *nif* promoter region
nifR1MR	ACAAGGTACCGATTACATTACACATATGTATCATGTTTCCGTT	
nifR2MF	TTTTCAAGAAAGGTTGGAGTCAGGTTAACATCGATGTCGGATG	EMSA probe of mutated GlnR-binding site II in the *nif* promoter region
nifR2MR	CATCCGACATCGATGTTAACCTGACTCCAACCTTTCTTGAAAA	
TS1F	ATACATATGTGTAATGTAATCTGACATTTGTTTTGACGA	SPR probe of GlnR-binding site I in the *nif* promoter region
TS1R	TCGTCAAAACAAATGTCAGATTACATTACACA	

	TATGTATCCTACCCTACGTCCTCCTGC	
TS2F	AAGGTTGGAGTCAGGTTAACTGACACGTCGGATGTAGGT	SPR probe of GlnR-binding site I in the *nif* promoter region
TS2R	ACCTACATCCGACGTGTCAGTTAACCTGACTCCAACCTTCCTACCCTACGTCCTCCTGC	
NSF	CAGTGAGGCACCTATCTCAGCGATCTGTCTCAGTGAGGC	Nonspecific DNA for SPR
NSR	GCCTCACTGAGACAGATCGCTGAGATAGGTGCCTCACTGCCTACCCTACGTCCTCCTGC	
pald1F1	GTAAGTCTTCCCAAGGAAAG	Identification of AdeR-binding site in the *ald1* promoter region by EMSA
pald1F2	TTTGAGGTAAGCATCGGTGA	
pald1F3	GATAAAACACAATCATGGCAG	
pald1F4	ATGCCCTTATCCCCAGGGAA	
pald1F5	AACTGCAGCGGGCATTTGC	
pald1F6	GAATGAAAAACTGACCTTCTC	
pald1R	CATTCTTCACATCTCCTG	
aldP1F	GAATGAAAAACTGACCTTCTCATTGTGATAAAACACAATC	EMSA probe of AdeR-binding site in the *ald1* promoter region
aldP1R	GATTGTGTTTTATCACAATGAGAAGGTCAGTTTTTCATTC	
aldP1MF	GAATGAAAAACTGACCTTGGATCCGTGATAAAACACAATC	EMSA probe of mutated AdeR-binding site in the *ald1* promoter region
aldP1MR	GATTGTGTTTTATCACGGATCCAAGGTCAGTTTTTCATTC	
**Construction of mutants with mutagenesis of the AdeR and GlnR-binding site(s)**		
adeRUF	ACGATGCGTCCGGCGTAGAGGATCCGGCGGCGTTGTCGGAAC	Mutation of AdeR-binding site in ald1 promoter
MAdeRR	TTTATCACGGATCCAAGGTCAGTTTTTCATTCAGC	
MAdeRF	GACCTTGGATCCGTGATAAAACACAATCATGGCAGA	
adeRDR	GCGACCACACCCGTCCTGTGGATCGGCCCATTGTATAGACC	
MRnif1	TAAAACGACGGCCAGTGCCAAGCTTCTATTTTCCGAGCTGCTTATC	Mutation of GlnR-binding site (s) in *nif* promoter
MRnif2	GTTCTCCGGCTTTATTGGCAATATATAATTGTTAGC	
MRnif3	TGCCAATAAAGCCGGAGAACCATAACGAAG	
MRnif4	GCGGCTTCATTTTTTCCCACCTCCTTAATGTAATG	
MRnif5	GTGGGAAAAAATGAAGCCGCAGCCGACG	
MRnif6	AGTCGACCTGCAGGCATGCAAGCTTTGCATCGAGGTCCACTACAGG	
